# Sex matters: Otolith shape and genomic variation in deacon rockfish (*Sebastes diaconus*)

**DOI:** 10.1002/ece3.5763

**Published:** 2019-11-07

**Authors:** Felix Vaux, Leif K. Rasmuson, Lisa A. Kautzi, Polly S. Rankin, Matthew T. O. Blume, Kelly A. Lawrence, Sandra Bohn, Kathleen G. O'Malley

**Affiliations:** ^1^ State Fisheries Genomics Lab Coastal Oregon Marine Experiment Station Department of Fisheries and Wildlife Hatfield Marine Science Center Oregon State University Newport OR USA; ^2^ Marine Resources Program Oregon Department of Fish and Wildlife Newport OR USA

**Keywords:** genotyping by sequencing, geometric morphometric, population genetic, sexual conflict, sexual dimorphism, stock assessment

## Abstract

Little is known about intraspecific variation within the deacon rockfish (*Sebastes diaconus*), a recently described species found in the northeast Pacific Ocean. We investigated population structure among fish sampled from two nearshore reefs (Siletz Reef and Seal Rock) and one offshore site (Stonewall Bank) within a <50‐km^2^ area off the Oregon coast. Fish from the three sample sites exhibited small but statistically significant differences based on genetic variation at >15,000 neutral loci, whether analyzed independently or classified into nearshore and offshore groups. Male and females were readily distinguished using genetic data and 92 outlier loci were associated with sex, potentially indicating differential selection between males and females. Morphometric results indicated that there was significant secondary sexual dimorphism in otolith shape, but further sampling is required to disentangle potential confounding influence of age. This study is the first step toward understanding intraspecific variation within the deacon rockfish and the potential management implications. Since differentiation among the three sample sites was small, we consider the results to be suggestive of a single stock. However, future studies should evaluate how the stock is affected by differences in sex, age, and gene flow between the nearshore and offshore environments.

## Graphical abstract

Otolith shape and genetic variation within the recently described Deacon Rockfish was investigated for the first time. Small but significant differences were detected among fish sampled from two nearshore reefs and one offshore site within a small geographic radius. Males and females differed in otolith shape and genetically which may indicate secondary sexual dimorphism and differential selection between the sexes, respectively.

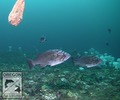

## 1 INTRODUCTION

A high diversity of *Sebastes* rockfish occur in the northeast Pacific Ocean (Hyde & Vetter, 2007; Love, Yoklavich, & Thorsteinson, 2002). Rockfish vary significantly in size, shape, and color, occupying most habitats, and species are an ecologically important component of many marine communities (Love et al., 2002). Rockfish support large and valuable commercial and recreational fisheries (Love et al., 2002). In Oregon, semipelagic nearshore rockfish are the primary target of the recreational fleet and these fisheries represent a vital economic component of coastal communities (Research Group, 2015a, 2015b). However, despite their economic importance, our understanding of the basic biology of these semipelagic species is lacking, which in turn affects our ability to conduct accurate stock assessments (Dick et al., 2017).

The deacon rockfish *Sebastes diaconus* Frable, Wagman, Frierson, Aguilar, and Sidlauskas (2015) was recently distinguished as a separate species from the blue rockfish *Sebastes mystinus* (Jordan & Gilbert, 1881). Although superficially similar, these species represent distinct genetic lineages and exhibit key phenotypic differences such as body coloration and cranial morphology (Burford, 2009; Burford & Bernardi, 2008; Burford, Carr, & Bernardi, 2011; Cope, 2004; Frable et al., 2015; Hannah, Wagman, & Kautzi, 2015). These species occur in sympatry from northern California to central Oregon; however, the deacon rockfish has a more northern distribution—extending to Vancouver Island, British Columbia, whereas the blue rockfish is more southern—reaching northern Baja California (Frable et al., 2015). Given the previous coupling of the two species, little is known about demographic, ecological, and genetic variation within deacon rockfish.

Previous studies investigating intraspecific variation in rockfish of the northeast Pacific have focused on the influence of the north–south latitudinal gradient on growth and maturity (Frey, Head, & Keller, 2015; Gertseva, Cope, & Matson, 2010). Clear breaks in population genetic structure have also been reported near oceanographic boundaries such as upwellings (Cope, 2004; Sivasundar & Palumbi, 2010). However, few studies have examined the influence of the east–west longitudinal gradient on intraspecific variation, which is closely related to depth change between the nearshore and offshore environments (Boehlert & Kappenman, 1980).

**Figure 1:**
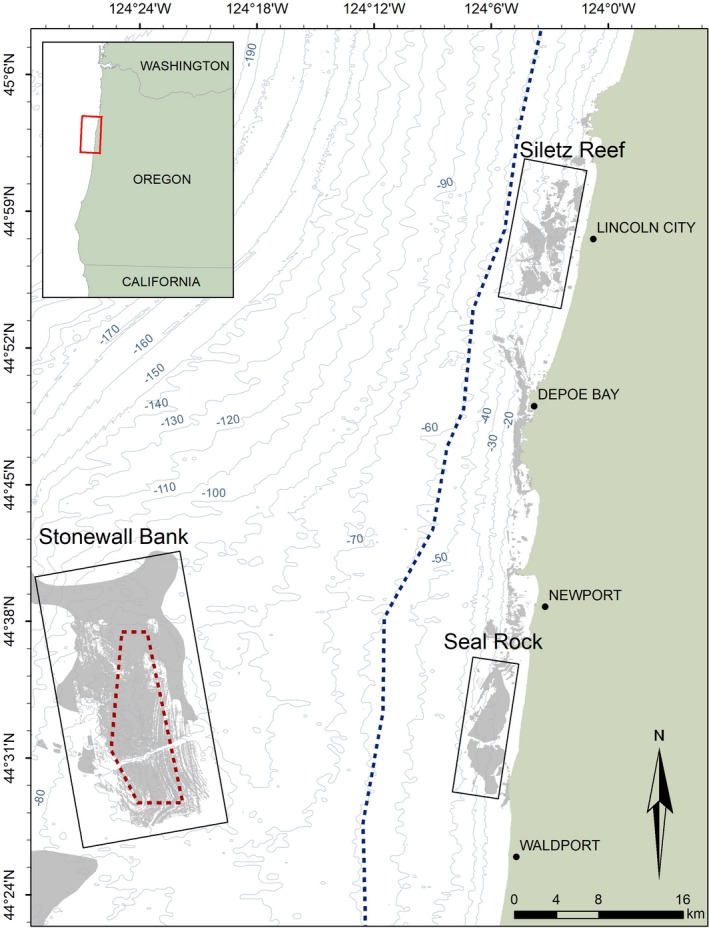
A map showing the three sample sites (Siletz Reef, Seal Rock, and Stonewall Bank) for deacon rockfish off the central Oregon coast. The blue dotted line illustrates the 55 m closure line for the rockfish fishery, and the red dotted line shows the Yelloweye Rockfish Conservation Area closure line at Stonewall Bank

Deacon rockfish inhabit a wide depth range, occurring from the shallow intertidal zone to depths >70 m (Frable et al., 2015; M. T. O. Blume unpublished data). Recent tagging research suggests that deacon rockfish have very small home ranges and do not migrate after settlement (P. S. Rankin, unpublished data). Deacon rockfish, along with other rockfish species, have been caught by commercial and recreational fishers in both the nearshore and offshore of the Oregon coast (Research Group, 2015a, 2015b). However, since 2003/2004, due to regulatory and economic reasons, depths ≥55 m have experienced little or no effort from both trawl (bottom and midwater) and fixed gear fisheries (recreational and commercial). In addition, to minimize catch of yelloweye rockfish *Sebastes ruberrimus* (Cramer, 1895), the Yelloweye Rockfish Conservation Area was established at Stonewall Bank in 2005, a previously heavily fished 10‐km‐long rocky outcrop that rises 20 m above the surrounding seafloor (Figure 1). As such, deacon rockfish at Stonewall Bank have been residing in a marine protected area since 2005 and most of the shelf has been in a *de facto* marine protected area since 2003/2004 (Figure 1). In 2017, fishing at depths ≥55 m opened to a new recreational fixed gear fishery that utilized modified terminal tackle (though deacon retention was prohibited until 2019) (Hannah, Buell, & Blume, 2008). Also in 2017, the markets for fish caught in the offshore midwater trawl fishery re‐emerged and the fishery began to operate again. In the nearshore, recreational and commercial hook and line fishing for deacon rockfish never closed and significant catches of deacon rockfish continue to occur.

Unlike the nearshore environment, life history data for deacon rockfish in offshore areas are limited due to the previous fishing restrictions. It is important to determine whether the nearshore and offshore represent distinct fish stocks so that assessment models can account for potential connectivity between the two areas. The need to precisely define stock boundaries in the management of rockfish was demonstrated by a recent population genetic study of three species sampled from Puget Sound and outer coastal waters (Andrews et al., 2018). Based on the genetic results, canary rockfish *Sebastes pinniger* (Gill, 1864) from Puget Sound and the outer coastal area were concluded to represent a single genetic population (Andrews et al., 2018). This result suggested that the species did not meet the criteria for Endangered Species Act listing, which was previously designated based on evidence from other rockfish species (Andrews et al., 2018).

Deacon rockfish are managed by the Pacific Fishery Management Council, the National Marine Fisheries Service, and each coastal state through measures such as annual catch limits for each stock or stock complex, harvest guidelines, trip or bag limits, area and gear restrictions, and seasonal closures. These measures are described in the Pacific Coast Groundfish Fishery Management Plan (PFMC, 2016). The most recent stock assessments combined deacon and blue rockfish (e.g., Dick et al., 2017), and it is uncertain how species differences influenced the stock assessment model. In Oregon, the stock boundaries were defined by the California state border to the south and the Washington border to the north.

The definition of a “fish stock” is ultimately a management decision (Carvalho & Hauser, 1994; Hilborn & Walters, 1992). Thus, for the purpose of this paper we follow Cadrin, Karr, and Mariani (2013) and define a fish stock as “an exploited fishery unit” that “may be a single spawning component, a biological population, a metapopulation, or comprise portions of these units. For management purposes, stocks are considered discrete units, and each stock can be exploited independently or catches can be assigned to the stock of origin.” Since demography and genetic variation are typically interlinked, fish stocks are often considered genetic populations (Coyle, 1998; Ovenden, Berry, Welch, Buckworth, & Dichmont, 2015; Waldman, 1999). However, variables such as practical limits related to fishing and phenotypic traits (e.g., length and age at maturity) can be of equal importance from a management perspective, and interdisciplinary approaches are therefore necessary to define fish stocks (Abaunza, Murta, & Stransky, 2013; Cadrin & Secor, 2009; Coyle, 1998; MacLean & Evans, 1981; Ovenden et al., 2015). Delineating stocks is important for the sustainable management of fish populations as it allows researchers to investigate the influence and interaction of environmental and anthropogenic factors (Begg, Friedland, & Pearce, 1999; Cadrin & Secor, 2009).

The use of variation in the shape of anatomical structures (e.g., otoliths and scales) to identify fish stocks has been used since Lea's (1929) seminal work on herring. The underlying assumption is that the shape of the structure reflects environmental differences among potential populations. These methods have matured since the advent of image processing methods and the implementation of Fourier transformations to analyze the outline of structures (Bird, Eppler, & Checkley, 1986; Castonguay, Simard, & Gagnon, 1991). Otolith shape has been used to identify and differentiate various *Sebastes* species (Christensen et al., 2018; Stransky & MacLellan, 2005; Zhuang, Ye, & Zhang, 2015), as well as stocks within some species (Stransky, 2005).

The integration of genetic information into fish stock assessments has been relatively slow, primarily because traditional genetic markers such as microsatellites typically provide limited insight toward recent population genetic change, or local adaptation in marine organisms (Waples, Punt, & Cope, 2008). The advent of high‐throughput sequencing methods has significantly increased the amount of data and the resolution of genetic insight for fisheries management in other species (Hauser & Carvalho, 2008; Kumar & Kocour, 2017; Riginos, Crandall, Liggins, Bongaerts, & Treml, 2016; Valenzuela‐Quiñonez, 2016). Many studies have attempted to identify neutral and adaptive genetic variation (e.g., Gagnaire et al., 2015; Nielsen, Hemmer‐Hansen, Foged Larsen, & Bekkevold, 2009; Ovenden et al., 2015; Valenzuela‐Quiñonez, 2016), which has improved the delineation of populations and fish stocks in both migratory species such as Greenland halibut *Reinhardtius hippoglossoides* (Walbaum, 1792) (Westgaard et al., 2017) and European hake *Merluccius merluccius* (Linnaeus, 1758) (Milano et al., 2014), and sedentary species such as bluespotted Cornetfish *Fistularia commersonii* Rüppell, 1838 (Bernardi, Azzurro, Golani, & Miller, 2016). Typically, neutral genetic variation reflects stochastic genetic drift and the degree of gene flow among populations, whereas adaptive variation suggests selective differences among populations (Funk, McKay, Hohenlohe, & Allendorf, 2012). Adaptive genetic variation can reflect differential selection on certain genes among populations, despite the absence of obvious genetic differentiation for other markers. In addition to neutral and adaptive genetic differences, it is important to consider genetic variation associated with sex (Grummer et al., 2019). Such variation and sex biases in sampling can lead to inaccurate interpretations of population genetic differentiation (Benestan et al., 2017), potentially leading to incorrect management decisions.

The aim of this study was to use an interdisciplinary approach (Abaunza et al., 2013) to test for population structure and potential fish stocks among deacon rockfish off the Oregon coast based on otolith shape and genetic variation. We sampled fish from two nearshore reefs (Siletz Reef and Seal Rock) and one offshore area (Stonewall Bank) within a small geographic radius (<50 km^2^). The three sample sites were analyzed independently, and differences between nearshore and offshore samples were tested as well. In order to assess the influence of sex in our analyses, we tested for otolith shape and genetic differences between males and females. To disentangle any potential interaction between sample location and sex, we also analyzed genetic variation among the three sample sites using males and females separately.

## 2 MATERIALS AND METHODS

### 2.1 Sampling

Deacon rockfish were collected from three sites located off the Oregon coast: Siletz Reef (44°59′N, 124°3′W), Seal Rock (44°33′N, 124°6′W), and Stonewall Bank (44°34′N, 124°25′W) (Figure 1). The two nearshore reefs, Siletz Reef and Seal Rock, occur at depths of 5–70 m and 12–64 m, respectively, whereas Stonewall Bank is an offshore reef with depths ranging from 44 to 117 m (Figure 1). The distance between Siletz Reef and Seal Rock is 42 km, and the distance between each site and Stonewall Bank is 46 km and 24 km, respectively (Figure 1).

Recreational hook and line gear was used for all collections. At each site, terminal gear included a variety of plastic baits, small‐ to medium‐sized flies, and Sabiki rigs (herring jigs). Prior efforts to collect deacon rockfish off Oregon have shown that Sabiki rigs are capable of capturing a wide size range of individuals (~8–40 cm in this study), which helped offset gear‐related bias in size selectivity of typical hook and line fishing gear (Ralston, 1990). At sea, fish were measured to total length and these data were used to ensure a wide range of size classes were sampled. No attempt was made to sex fish at sea. Fin clips for genetics were also taken at sea (see below for methods), and then, whole fish were placed on ice until later dissection of otoliths.

Otoliths were sampled from 676 fish, with 110, 172, and 394 specimens from Siletz Reef, Seal Rock, and Stonewall Bank, respectively (Table 1A). Sampling was conducted between December 2016 and November 2017 during favorable weather periods. At the selected sample areas, a total of 50 individuals were collected every month except January, June, and September (*n* = 9 per area). Sampling efforts each month were mostly constrained to a 24‐hr period, although low catch rates at Seal Rock meant that fish collected on August 8th and 16th 2017 were combined to achieve adequate sampling. Age was determined for all otolith samples using the break and burn method (Chilton & Beamish, 1982; Figure S1).

**Table 1:** Deacon rockfish sampled for otolith shape and genetic analyses

(A) Otoliths					
Sites	Male	Female	Unidentified	Site totals	Group totals
Siletz Reef	39	66	5	110	282 (nearshore)
Seal Rock	58	111	3	172	
Stonewall Bank	134	260	0	394	394 (offshore)
Total	676 †668				

(B) Genetic three sample sites				
Sites	Male	Female	Unidentified	Site totals
Siletz Reef	7	16	2	25
Seal Rock	6	17	0	23
Stonewall Bank	9	16	0	24
Total	73			

(C) Genetic nearshore versus offshore				
Groups	Male	Female	Unidentified	Group totals
Nearshore	13	33	2	48
Offshore	13	35	0	48
Total	96 †94			

(D) Genetic female‐only three sample sites	
Sites	Female
Siletz Reef	16
Seal Rock	17
Stonewall Bank	17
Total	50

(E) Genetic male‐only three sample sites	
Sites	Male
Siletz Reef	7
Seal Rock	6
Stonewall Bank	7
Total	20

Note

Sampling is listed for each sample site (Siletz Reef, Seal Rock, and Stonewall Bank), for the tested nearshore and offshore groups, and for males (♂), females (♀), and individuals of unknown sex (U). The dagger sign (†) indicates where sample sizes were reduced as the sex of a small number of individuals was unknown. A full list of specimens used for each RAD sequencing dataset is provided in Table S1, and a spreadsheet in the online supplement lists all otolith samples. The datasets are as follows: (A) Otolith dataset, used to analyze shape variation among the three sample sites (*N* = 676), between the nearshore and offshore groups (*N* = 676), and between males and females (*n* = 668). (B) Genetic dataset for variation among the three sample sites (*n* = 73). (C) Genetic dataset for variation between the nearshore and offshore groups (*N* = 96), and between males and females (*n* = 94). (D) Genetic dataset for variation among the three sample sites using only females (*n* = 50). (E) Genetic dataset for variation among the three sample sites using only males (*n* = 20).

Funding allowed a total of 96 fish to be sampled for genetic analysis, with 25 and 23 nearshore specimens from Siletz Reef and Seal Rock, respectively, and 48 offshore individuals from Stonewall Bank (Table 1C). All genetic samples from Siletz Reef and Seal Rock were collected within a single sampling effort on October 4th and November 6th, respectively. Fish from Stonewall Bank were collected in two even efforts on October 5th and November 6th.

### 2.2 Otolith shape digitization and analysis

Fish were stored on ice for up to 24 hr, and otoliths were extracted using forceps after cutting the cranium. Otoliths were rinsed with freshwater, air‐dried, and stored in binned otolith trays. All otoliths were used to investigate morphometric variation among the three sample sites and between the nearshore and offshore groups (*N* = 676; Table 1A). The sex of some smaller individuals (*n* = 8) was indeterminable; therefore, a slightly smaller dataset was used to estimate otolith shape differences between males and females (*n* = 668; Table 1A).

Images of sagittal otoliths were taken using a Leica DFC 290 camera mounted on a Leica MZ 9.5 optical microscope. Otoliths were placed on black fabric, sulcus side down, and oriented with the rostrum to the left. All otoliths, as well as a metric ruler for scaling, were imaged at 0.63× magnification. For consistency, only the left sagittal otolith of each individual was selected, except for a small number of individuals (*n* = 8) where the left otolith was damaged or unavailable and the right otolith was used instead. Right side otolith images were horizontally transformed using adobe photoshop CS6 to correct the orientation in the digitization process. The nonparametric PERMANOVA test implemented in the shaper 0.1‐5 R package (Libungan & Pálsson, 2015; R Core Team, 2018) did not find any significant differences between the right and left otoliths of the sampled deacon rockfish, suggesting that differences observed among groups in this study were unlikely to be influenced by fluctuating asymmetry. A similar ANOVA method was used to estimate fluctuating asymmetry in an otolith shape analysis of lutjanid fishes (Vignon, 2015). Images were scale calibrated using fiji 1.51w (Schindelin et al., 2012).

Otolith shape was analyzed using shaper. The same method was previously used to distinguish two *Sebastes* species with high accuracy, but intraspecific variation was not investigated (Christensen et al., 2018). Contours were automatically extracted, and shape coefficients were estimated using a wavelet transformation. shaper implements both Fourier and wavelet transformations, and a comparison of the results from either transformation did not result in significantly different results. We decided to use the wavelet transformation. The shape coefficients were then standardized for fish length using the methods of Lleonart, Salat, and Torres (2000) that are implemented in the shaper package, with the aim of controlling for size and potential ontogenetic differences among fish of different ages. Variation among the potential populations was analyzed using a PERMANOVA test, using default settings with 1,000 permutations (Libungan & Pálsson, 2015). No interactions were tested due to the fact that area and sex were confounding. Otolith shape variation among samples was visualized using a canonical analysis of principal coordinates (CAP; Anderson & Willis, 2003) in the vegan 2.5‐2 R package (Oksanen et al., 2018).

The effect of sample size on the accuracy of the PERMANOVA test and CAP was investigated. We randomly subsampled, with replacement, the dataset of 676 otoliths 1,000 times and generated datasets increasing in sample size by multiples of 50 up to 500 (i.e., 50, 100, 150, … 500). Each dataset was divided evenly between sampling from the nearshore and offshore areas, and we tested the discrimination of those groups.

### 2.3 DNA extraction and sequencing

A piece of caudal fin was taken from each fish and stored in a 5‐ml vial filled with 95% ethanol. Whole genomic DNA was extracted following the protocol and buffer solutions described by Ivanova, Dewaard, and Hebert (2006). DNA was quantified using Qubit high‐sensitivity dsDNA fluorometric quantitation (Life Technologies, Thermo Fisher Scientific Inc.).

All available DNA samples were used to analyze variation between the potential nearshore and offshore populations (*N* = 96; Table 1C), and males and females (*n* = 94; Table 1C). The three sample sites were analyzed independently with approximately the same number of samples per site (*n* = 73; Table 1B). Two further subsampled datasets were used to analyze variation among the three sample sites using only males (*n* = 20; Table 1D) and only females (*n* = 50; Table 1E).

100 ng DNA was prepared for restriction site‐associated DNA sequencing (RADseq). Genomic DNA was digested with *SbfI‐HF* restriction enzyme (low frequency, 8 bp cutter, 5′…CCTGCAGG… 3′; New England Biolabs, Inc.). DNA extractions for 96 deacon rockfish individuals, as well as six quality control repeats of specimens (1 from Seal Rock, 2 from Siletz Reef, 3 from Stonewall Bank), were organized randomly across three 96‐well sequencing plates (Table S1). Each plate also included a negative control without any template DNA. All three library plates were run on an Illumina HiSeq 3000 lane using 150‐bp paired‐end sequencing chemistry at Oregon State University's Center for Genome Research and Biocomputing.

### 2.4 Processing of RAD sequencing data

A total of 342,702,104 DNA read pairs were sequenced (Table S2). stacks 1.47 (Catchen, Amores, Hohenlohe, & Postlethwait, 2011; Catchen, Hohenlohe, Bassham, Amores, & Cresko, 2013) was used to process reads, identify loci, and estimate genotypes. Forward and reverse reads from each index were demultiplexed into separate inline barcodes using the *process_radtags* component of the stacks pipeline. Simultaneously, the *process_radtags* step was used to remove reads with low‐quality read data and ambiguous barcodes and RAD tags, resulting in a total of 291,066,365 read pairs being retained (Table S2). This step included the rescue barcode and RADtag parameter (‐r) to retrieve additional reads. Only single‐end (forward, R1) reads containing the *SbfI* restriction site were analyzed downstream.

Reads were assembled into stacks of similar DNA sequences and then into catalogs of reads for each investigated dataset using *ustacks* and *cstacks*. Following the recommendation of Mastretta‐Yanes et al. (2015), many of the parameters in *ustacks*, *cstacks,* and *populations* were modified to examine the RAD sequencing data comprehensively (Table S3). In *ustacks* and *cstacks*, the default parameters (‐m 2 ‐M 2 ‐N 4 ‐n 1) used in our final analyses were judged to provide a high number of stacks, consistent with other parametrizations (Table S3), with a low risk to introducing a high rate of erroneous reads. In *ustacks*, this meant that the minimum depth of coverage used to create a stack was two (‐m 2), the maximum distance (in nucleotides) allowed between stacks was two (‐M 2), and the maximum distance allowed to align secondary reads to primary stacks was four (‐N 4). A bounded SNP model was applied with the error rate not being allowed to exceed 5% (‐‐bound_high 0.05). In *cstacks*, the number of mismatches allowed between sample loci when building a catalog was one (‐n 1). Locus coverage depth per individual was similar across the tested datasets, although some individuals yielded more loci with adequate coverage than others. As an example, Figure S2 presents mean (with standard deviation) and maximum coverage depth per individual for the dataset comparing nearshore and offshore fish (*N* = 96), where the overall mean coverage depth was 18.7 reads (*SD* 12.2, mean maximum 95.1).

Population genetic variation was estimated using the *populations* component of the stacks pipeline. In *populations*, the minimum stack depth required for individuals at a locus was set at five (‐m 5). Samples were organized into multiple, independent datasets, which differed in the number of individuals and designated populations used to construct a loci catalog (Table 2, Table S1). The datasets were three independent sample sites, nearshore versus offshore, male versus female, female‐only three sample sites, and male‐only three sample sites. The minimum number of populations that a locus needed to be present in (‐p) was set to the same number of populations set for each dataset (e.g., nearshore vs. offshore ‐p 2; three sample sites ‐p 3; sex ‐p 2). The minimum percentage of individuals in a population required to process a locus for a given population was set at 60% (‐r 0.6). A minimum allele frequency of 5% was enforced for loci (‐‐min_maf 0.05). Only the first SNP of each locus was included (‐‐write_single_snp). All variant SNPs were biallelic.

**Table 2:** The deacon rockfish loci datasets (labeled A–E) produced from the *populations* component of stacks 1.47

# ind.	# pop.	# PSVs removed	# loci in LD removed	# loci out of HWE	# final variant loci
(A) Three sample sites					
73	3	314	7	0	15,371
(B) Nearshore versus offshore					
96	2	329	0	0	15,937
(C) Male versus female					
94	2	336	0	0	15,657
(D) Female‐only three sample sites					
50	3	268	0	0	14,678
(E) Male‐only three sample sites					
20	3	640	115	0	14,564

Note

Loci estimated to be paralogous sequence variants (PSVs) and in linkage disequilibrium (LD) were removed. No loci were identified to be out of Hardy–Weinberg equilibrium (HWE). For an exact list of individuals included in each dataset, see Table S1.

Putative paralogous sequence variants (PSV) were identified using the python and R scripts for paralog‐finder 1.0 (Mortiz, 2018), which is based on hdplot (McKinney, Waples, Seeb, & Seeb, 2017) and accounts for varying degrees of missing data per locus. Loci estimated to be in linkage disequilibrium (LD) were identified using plink 1.9 (Purcell et al., 2007). Putative PSVs and one locus for each loci pair estimated to be in LD were organized into a blacklist (‐B; Catchen et al., 2013), and the *populations* component of stacks was rerun (same settings as above) so that these sites were removed from subsequent analyses (Table 2). We tested for conformance to Hardy–Weinberg equilibrium (HWE) using vcftools 0.1.16 (Danecek et al., 2011; Table 2). This HWE estimation used an exact test (Wigginton, Cutler, & Abecasis, 2005), and we corrected for multiple testing by using a false discovery rate (FDR) adjustment for *p*‐values with a critical threshold of <5% (Allendorf, Luikart, & Aitken, 2013; Bouaziz, Jeanmougin, & Geudj, 2012; Storey, 2002; Waples, 2015). The format of output files from stacks was converted for analyses in downstream software using pgdspider 2.1.1.3 (Lischer & Excoffier, 2012).

### 2.5 Genetic variation

After removing loci estimated to be putative PSVs or in LD, we estimated observed (*H*_O_) and expected heterozygosity (*H*_E_) for each group tested in the separate loci datasets, using the adegenet 2.1.1 R package (Jombart, 2008; Jombart & Ahmed, 2011). Using the same R package, we also estimated allelic richness and the inbreeding coefficient (*F*_IS_) for each tested group. The whoa 0.01 R package was used to investigate genotype frequencies, as well as the relationship between read depth per locus and heterozygote miscall rates (Anderson, 2018). The level of relatedness among individuals was assessed using the Wang relatedness estimator implemented in coancestry 1.0.1.9 (Wang, 2011). The Wang relatedness estimator is appropriate for small sample sizes (<50 individuals) with many loci (Wang, 2017).

Genetic variation among sampled individuals and groups was explored using principal components analysis (PCA), again implemented in adegenet (Jombart, 2008; Jombart & Ahmed, 2011). For PCA, outlier loci were not removed but rather all loci within each dataset were analyzed together. We determined the number of “meaningful” principal components (PCs) to retain for interpretation and downstream analyses by using the broken‐stick test on PC Eigen values in the vegan R package. Retained PCs are axes that explain more variance among samples than expected by chance alone (Cangelosi & Goriely, 2007; Jackson, 1993).

### 2.6 Outlier loci and genetic differentiation

Outlier loci were estimated independently using four genome scan programs: fsthet 1.01 (Flanagan & Jones, 2017a), outflank 0.2 (Whitlock & Lotterhos, 2015), bayescan 2.1 (Foll & Gaggiotti, 2008), and pcadapt 4.0.3 (Luu, Bazin, & Blum, 2017). Four separate programs were used because the stringency of outlier classification, false discovery rate, and the fit of applied models to particular patterns of genetic variation are known to vary among methods (see discussion Ahrens et al., 2018; Flanagan & Jones, 2017a; Luu et al., 2017; Narum & Hess, 2011). Outlier loci identified by fsthet, outflank, and bayescan are *F*_ST_ outliers, which are sites that exhibit higher genetic differentiation among groups than expected by a neutral model (Ahrens et al., 2018; Foll & Gaggiotti, 2008). However, these programs use different statistical methods. fsthet analyzes the empirical relationship between *F*_ST_ and observed heterozygosity (Flanagan & Jones, 2017a), whereas outflank analyzes the distribution of a special form of *F*_ST_ that does not correct for sample size (Whitlock & Lotterhos, 2015). bayescan uses Bayesian maximum‐likelihood to analyze differences in allelic frequencies among groups (Foll & Gaggiotti, 2008). In contrast, pcadapt does not consider *F*_ST_, and instead, loci are identified as outliers with respect to population structure among sampled individuals, using PCA (Luu et al., 2017).

Default settings were used for fsthet and outflank (Flanagan & Jones, 2017a; Whitlock & Lotterhos, 2015). In bayescan, we used default parameters and a prior of 100, with a *q*‐value threshold of 0.05 (analogous to an FDR of 5%; Foll & Gaggiotti, 2008), and output data were investigated using the boa 1.1‐8‐2 R package (Smith, 2007). In pcadapt, we applied an *α* value of 0.05 (default = 0.1) to estimate outliers, alongside otherwise default settings, and the number of PCs used for each analysis (*K*) was determined using the aforementioned broken‐stick test on PC Eigen values. The qvalue 2.12.0 R package was used to estimate FDR for pcadapt (Storey, Bass, Dabney, & Robinson, 2018). Given the underlying assumptions of pcadapt (Luu et al., 2017), outlier detection results from the program were treated as negative (no outlier loci) if there was no obvious population structure for any PC. *F*_ST_‐based genome scan methods are known to be less accurate for comparisons of two groups (Flanagan & Jones, 2017a; Whitlock & Lotterhos, 2015) and may require larger sample sizes than comparisons of multiple populations (Foll & Gaggiotti, 2008; Whitlock & Lotterhos, 2015). As a conservative precaution against Type I error, only loci identified as outliers by all four programs were organized into separate outlier loci datasets. Without information from annotated genome or selection studies, we interpret all loci as putatively adaptive and presumed neutral (Shafer et al., 2015), and it is not yet possible to exclude the potential influence of genetic incompatibilities or genetic surfing upon observed allelic frequencies (Bierne, Welch, Loire, Bonhomme, & David, 2011; Excoffier & Ray, 2008).

We examined genetic differentiation among the groups tested in all five loci datasets, including the comparison of the three sample sites, the nearshore and offshore groups, and males and females. Neutral and outlier loci were analyzed independently using Weir and Cockerham's (1984) pairwise fixation index (*F*_ST_), implemented in the stampp 1.5.1 R package using 5,000 bootstraps (Pembleton, Cogan, & Forster, 2013). We included an FDR adjustment for *p*‐values with a critical threshold of <5% for the *F*_ST_ *p*‐values, using the same method as used for HWE estimation.

Genetic population structure was investigated among groups using Bayesian genotypic clustering in structure 2.3.4 (Pritchard, Stephens, & Donnelly, 2000). We tested for up to five potential genotypic clusters among individuals (*K* = 1–5). For each value of *K*, five replications of the admixture model with independent allele frequencies were applied, with an MCMC length of 50,000 generations and a 10% burn‐in. The optimal number of clusters was determined by examining estimates of mean *K* probability for a given value of *K* (Pritchard et al., 2000), and deltaK, the rate of change in logarithmic probability of the data (Evanno, Regnaut, & Goudet, 2005) implemented in structure harvester 0.6.94 (Earl & vonHoldt, 2012).

### 2.7 Identity of outlier loci

The genomic identity of outlier loci was investigated by exporting the 143‐bp FASTA format consensus sequence of each outlier locus from the stacks catalog and using NCBI blastn (Altschul, Gish, Miller, Myers, & Lipman, 1990) to align outlier loci with sequences available on GenBank. To investigate the identity of any outlier loci associated with sex, we conducted a pairwise alignment between outliers and the 26 male‐specific, sex‐linked loci identified for black‐and‐yellow *Sebastes chrysomelas* (Jordan & Gilbert, 1881) and gopher rockfish *Sebastes carnatus* (Jordan & Gilbert, 1880) by Fowler and Buonaccorsi (2016).

Using a modified approach of Fowler and Buonaccorsi (2016), we investigated whether our outliers occurred on the same chromosome. First, we used the BWA‐MEM algorithm in bwa 0.7.12 (Li & Durbin, 2009) to map outlier loci to an unannotated, representative scaffold genome of the flag rockfish *Sebastes rubrivinctus* (Jordan & Gilbert, 1880) (RefSeq: GCA_000475215.1). This species was used as it the closest available relative to deacon rockfish (Hyde & Vetter, 2007; Li, Gray, Love, Asahida, & Gharrett, 2006). Second, blat (Kent, 2002) was used to map scaffolds, trimmed to the first 25,000 bp (maximum length for blat), of the flag rockfish against annotated genomes of several other fish species: three‐spined stickleback *Gasterosteus aculeatus* (Linnaeus, 1758) (RefSeq: GCA_000180675.1)*,* fugu *Takifugu rubripes* (Temminck & Schlegel, 1850) (RefSeq: GCF_000180615.1)*,* and Japanese medaka *Oryzias latipes* (Temminck & Schlegel, 1846) (RefSeq: GCF_002234675.1).

## 3 RESULTS

### 3.1 Otolith shape analysis

**Figure 2:**
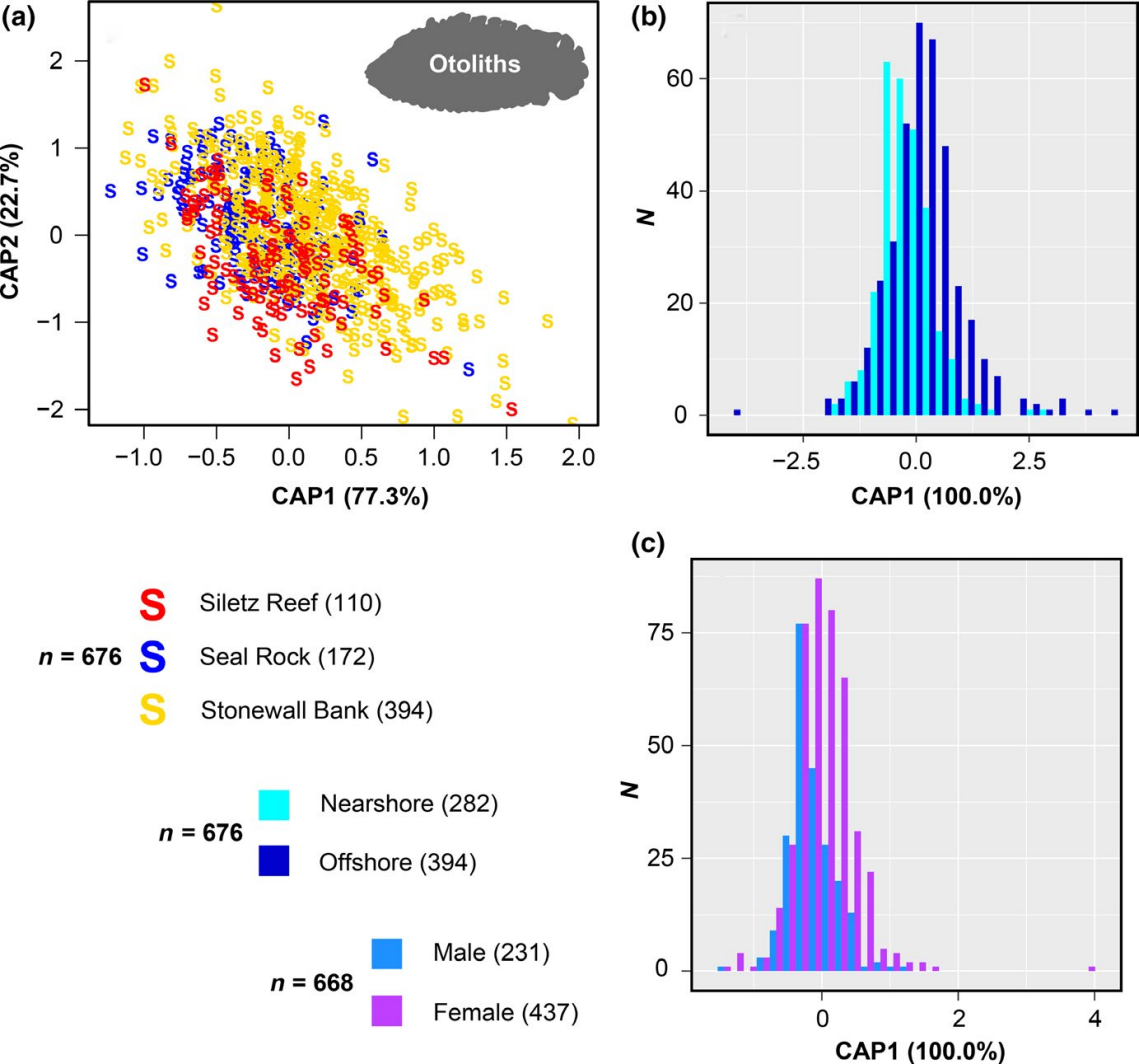
Scatterplots presenting otolith shape variation estimated by canonical analysis of principal coordinates (CAP) for comparisons of potential populations of deacon rockfish. (a) Comparing the three sample sites: Siletz Reef (*n* = 110), Seal Rock (*n* = 172), and Stonewall Bank (*n* = 394). The two CAP axes represented 77.3% and 22.7% of variation among individuals. A silhouette of an otolith from the species is illustrated. (b) Comparing fish from the nearshore and offshore: nearshore (Siletz Reef and Seal Rock, *n* = 282) and offshore (Stonewall Bank, *n* = 394). One CAP axis represented 100% of variation among individuals. (c) Comparing males and females: males (*n* = 231) and females (*n* = 437). One CAP axis represented 100% of variation among individuals

The PERMANOVA test found statistically significant differences in otolith shape between groups (nearshore and offshore) and among sites (Siletz, Seal Rock, and Stonewall Bank; Table 3A,B). The pseudo *F*‐values for these comparisons were similar (3.1–3.9; Table 3A,B), indicating otolith shape differences among Siletz Reef, Seal Rock, and Stonewall Bank were similar when analyzed independently, or organized into nearshore and offshore groups. Using CAP to visualize shape variation among individuals, it appears that all sites could be distinguished, despite substantial overlap (Figure 2). In the CAP, otolith shape variation among the three sample sites was differentiated using two axes, whereas variation between the nearshore and offshore groups used only one axis (Figure 2).

**Table 3:** Results from the PERMANOVA tests used to analyze otolith shape differences between the potential deacon rockfish populations

	Comparison	*n*	DF	Variance	Var. residual	pseudo *F*‐value	*p*
A	Three sample sites	676	2	0.0035	0.2963	3.9663	.001*
B	Nearshore versus offshore	676	1	0.0026	0.4549	3.8637	.004*
C	Male versus female	668	1	0.0087	0.4784	11.1980	.001*

Note

Tested datasets are labeled A–C.

An asterisk (*) indicates statistically significance.

There was a significant otolith shape difference between males and females, with a larger pseudo *F*‐value compared to the differences among sample sites (11.2; Table 3C). Graphs produced by CAP indicated that males and females exhibited distinctive distributions, but there was considerable overlap between them (Figure 2). Variation between males and females was restricted to a single axis (Figure 2).

In the investigation of potential sample size effects, probability density plots revealed that the central tendency was relatively consistent as sample size increased for both the nearshore and offshore groups (Figure S3). However, CAP plots for the discrimination of the two groups showed that the distinction of the groups increased significantly as sample size increased from 50 to 350 individuals (Figure S4). After 350 individuals, however, increased sample size did not appear to have a significant effect upon discrimination (Figure S4), and the mean value of the first CAP axis experienced only minor changes (Figure S5). Results from the PERMANOVA test also indicated that the average *F*‐statistic changed little after sampling ≥350 individuals (Figure S5).

### 3.2 Genetic variation

After controlling for multiple testing, no loci showed significant deviation from Hardy–Weinberg equilibrium (Table 2). We removed loci likely to be PSVs or in LD from our datasets (Table 2). The number of loci in each dataset was similar (~14,000–16,000), despite the varying number of individuals and groups (Table 2).

Across all five datasets, values for observed and expected heterozygosity were similar, with slightly fewer heterozygotes observed than expected (average difference across datasets of 0.02; Table 4). This slight deficiency in heterozygotes was reflected with positive *F*_IS_ values (Table 4). This result could be due to genotyping error or the sampling of a higher number of related individuals than expected by chance. Based on analyses in whoa (Anderson, 2018), genotypic frequencies did not appear to be significantly biased and an increased heterozygote miscall rate for loci with low read coverage is unlikely to have significantly affected the results (Figures S6–S8). According to the Wang relatedness estimator, however, we found no evidence for high relatedness (≥0.25) within and among the three sample sites using 73 individuals (Table S4). Allelic richness was also similar among groups tested in each dataset (Table 4).

**Table 4:** Genetic summary statistics for each dataset (labeled A–E) including observed (*H*_O_) and expected heterozygosity (*H*_E_), the inbreeding coefficient (*F*_IS_), and allelic richness (AR)

# indiv.	# pop.	# loci	Estimate	Tested populations						
				Siletz Reef	Seal Rock	Stonewall Bank	Nearshore	Offshore	Male	Female
(A) Three sample sites										
73	3	15,371	*H*_O_	0.2714	0.2647	0.2709				
			*H*_E_	0.2847	0.2846	0.2849				
			*F*_IS_	0.0466	0.0698	0.0492				
			AR	1.96	1.96	1.96				
(B) Nearshore versus offshore										
96	2	15,937	*H*_O_				0.2681	0.2715		
			*H*_E_				0.2862	0.2866		
			*F*_IS_				0.0487	0.0384		
			AR				1.99	1.99		
(C) Males versus females										
94	2	15,657	*H*_O_						0.2733	0.2678
			*H*_E_						0.2845	0.2852
			*F*_IS_						0.0393	0.0611
			AR						1.97	1.97
(D) Female‐only three sample sites										
50	3	14,678	*H*_O_	0.2700	0.2655	0.2684				
			*H*_E_	0.2831	0.2842	0.2843				
			*F*_IS_	0.0465	0.0661	0.0558				
			AR	1.92	1.92	1.92				
(E) Male‐only three sample sites										
20	3	14,564	*H*_O_	0.2446	0.2351	0.2472				
			*H*_E_	0.2545	0.2546	0.2576				
			*F*_IS_	0.0388	0.0766	0.0404				
			AR	1.67	1.67	1.67				

**Figure 3:**
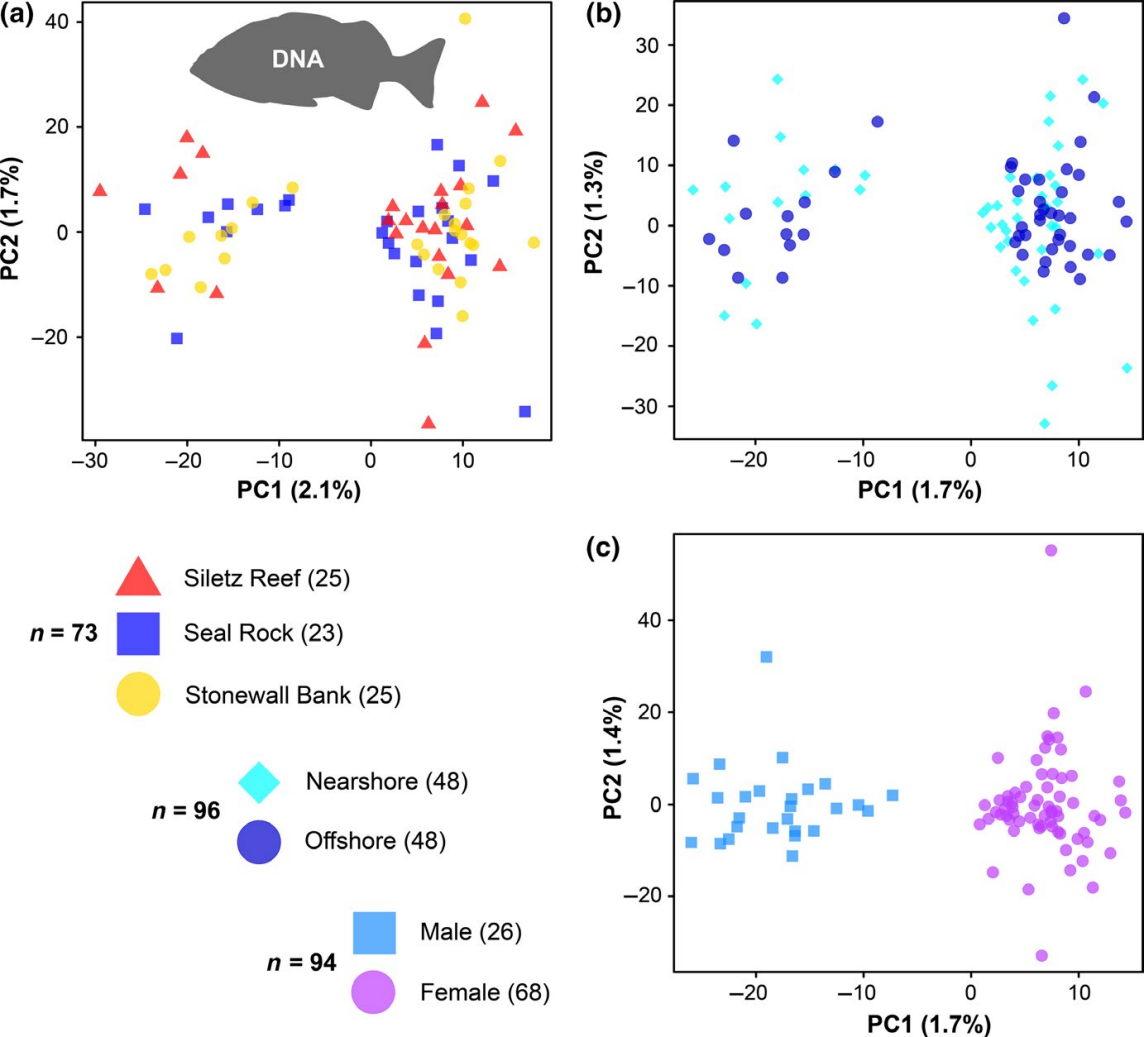
Scatterplots presenting genetic variation among deacon rockfish as estimated by principal components analysis (PCA), based on the SNP genotype data for all loci in each dataset. Plots show results for each dataset: (a) three sites, (b) two groups, and (c) sex. Sample sizes and the coloration of individuals are explained in the key, although it should be noted that PCA only visualizes the variance among samples. The first two PCs presented represent 2.1% and 1.7% of variation among individuals in (a), 1.7% and 1.3% of variation in (b), and 1.7% and 1.4% of variation in (c)

No obvious population structure was revealed by PCA in comparisons of the three sample sites or the nearshore and offshore groups, and all PCs failed the broken‐stick test—meaning that any patterns observed were likely to be a product of chance. However, in the comparisons of males and females, it was obvious that the first PC in each dataset reflected sex (Figure 3a,b). This suspicion was confirmed in the dataset comparing males and females (Figure 3c), where the separation across PC1 was clearly associated with sex. When genetic variation was examined among the three sample sites using only males or only females, PC1 (and all further PCs) for each dataset no longer exhibited any obvious structure (Figure S9).

### 3.3 Outlier loci and population genetic differentiation

No outlier loci were identified by all four genome scan programs (fsthet, outflank, bayescan, or pcadapt) when comparing the three sample sites or the nearshore and offshore groups (Table 5). In contrast, 92 outlier loci were identified by all four genome scan programs for the dataset comparing males and females (Table 5c). Comparisons of *F*_ST_ and observed heterozygosity (*H*_O_) for each locus in the sex comparison dataset, as used by fsthet, are shown in Figure S10.

**Table 5:** Identifying outlier (putatively adaptive) loci in the RAD sequencing datasets using the genome scan programs fsthet, outflank, bayescan, and pcadapt

# ind.	# pop.	total # loci	# outlier loci estimated				Final datasets	
			fsthet	outflank	bayescan	pcadapt	# putatively adaptive	# presume d neutral
(A) Three sample sites								
73	3	15,371	637	1	0	0	0	15,371
(B) Nearshore versus offshore								
96	2	15,937	415	8	0	0	0	15,937
(C) Male versus female								
94	2	15,657	417	221	93	197	92	15,565
(D) Female‐only three sample sites								
50	3	14,678	875	0	0	0	0	14,678
(E) Male‐only three sample sites								
20	3	14,564	1,146	0	0	0	0	14,564

Using presumed neutral loci, all pairwise *F*_ST_ estimates were statistically significant for comparisons of the three sample sites and between the nearshore and offshore groups (Table 6A,B). However, the estimated *F*_ST_ values for these neutral loci were low, ranging from 0.0004 to 0.0013 (Table 6A,B). We found evidence for statistically significant *F*_ST_ differences between males and females based on both neutral and outlier loci (Table 6C). The *F*_ST_ value based on neutral loci was 0.0036 (0.0030–0.0042, *p* < .0001), whereas the *F*_ST_ value estimated for outlier loci was much higher at 0.45 (0.4204–0.4697, *p* < .0001; Table 6C). Removing outlier loci associated with sex did not impact the *F*_ST_ estimates for the site comparisons (Table S5).

**Table 6:** Pairwise *F*_ST_ values (Weir & Cockerham, 1984) for each of the five datasets, using the stampp R package

# ind.	# pop.	Loci dataset	# loci	Estimate	Siletz Reef versus Seal Rock	Siletz Reef versus Stonewall Bank	Seal Rock versus Stonewall Bank	Nearshore versus Offshore	Male versus Female
(A) Three sample sites									
73	3	Presumed neutral	15,371	*F*_ST_	0.0013	0.0009	0.0010		
				95% CI	(0.0006–0.0020)	(0.0002–0.0015)	(0.0003–0.0016)		
				*p*‐value	<0.0001	0.0038	0.0008		
				Signif.	*	*	*		
(B) Nearshore versus offshore									
96	2	Presumed neutral	15,937	*F*_ST_				0.0004	
				95% CI				(0.0001–0.0008)	
				*p*‐value				0.004	
				Signif.				*	
(C) Male versus female									
94	2	Presumed neutral	15,565	*F*_ST_					0.0036
				95% CI					(0.0030–0.0042)
				*p*‐value					<0.0001
				Signif.					*
94	2	Putatively adaptive	92	*F*_ST_					0.45
				95% CI					(0.4204–0.4697)
				*p*‐value					<0.0001
				Signif.					*
(D) Female‐only three sample sites									
50	3	Presumed neutral	14,678	*F*_ST_	0.0017	0.0012	0.0005		
				95% CI	(0.0006–0.0027)	(0.0002–0.0022)	(−0.0005 to 0.0015)		
				*p*‐value	0.0006	0.0118	0.1562		
				Signif.	*	*			
(E) Male‐only three sample sites									
20	3	Presumed neutral	14,564	*F*_ST_	0.0021	0.0036	0.0000		
				95% CI	(−0.0002 to 0.0045)	(0.0013–0.0060)	(−0.0025 to 0.0024)		
				*p*‐value	0.0406	0.0006	0.5342		
				Signif.		*			

Note

Estimated 95% confidence intervals (in parentheses) and *p*‐values are listed below each *F*_ST_ estimate (using 5,000 bootstraps). Statistically significant results are marked with an asterisk (*).

For the female‐only dataset comprising the three sample sites (*n* = 50), we found statistically significant differences for two out of three pairwise comparisons based on variation at the neutral loci (Table 6D). Siletz Reef was significantly different from both Seal Rock (*F*_ST_ = 0.017, *p* = .0006) and Stonewall Bank (*F*_ST_ = 0.0012, *p* = .0118), but Seal Rock was not significantly different from Stonewall Bank (*F*_ST_ = 0.0005, *p* = .1562; Table 6D). For the male‐only dataset comprising the three sample sites (*n* = 20), one out of the three pairwise comparisons was significant. Again, Siletz Reef was significantly different from Stonewall Bank (*F*_ST_ = 0.0036, *p* = .0006), but Siletz Reef and Seal Rock (*F*_ST_ = 0.0021, *p* = .0406) and Seal Rock and Stonewall Bank were not significantly different (*F*_ST_ = 0.0000, *p* = .5342; Table 6E). Although significance varied in these smaller datasets, *F*_ST_ values indicated that the degree of genetic differentiation among males or females from the three sample sites was similar to the results in the three sample site dataset that included both sexes (Table 6).

**Figure 4:**
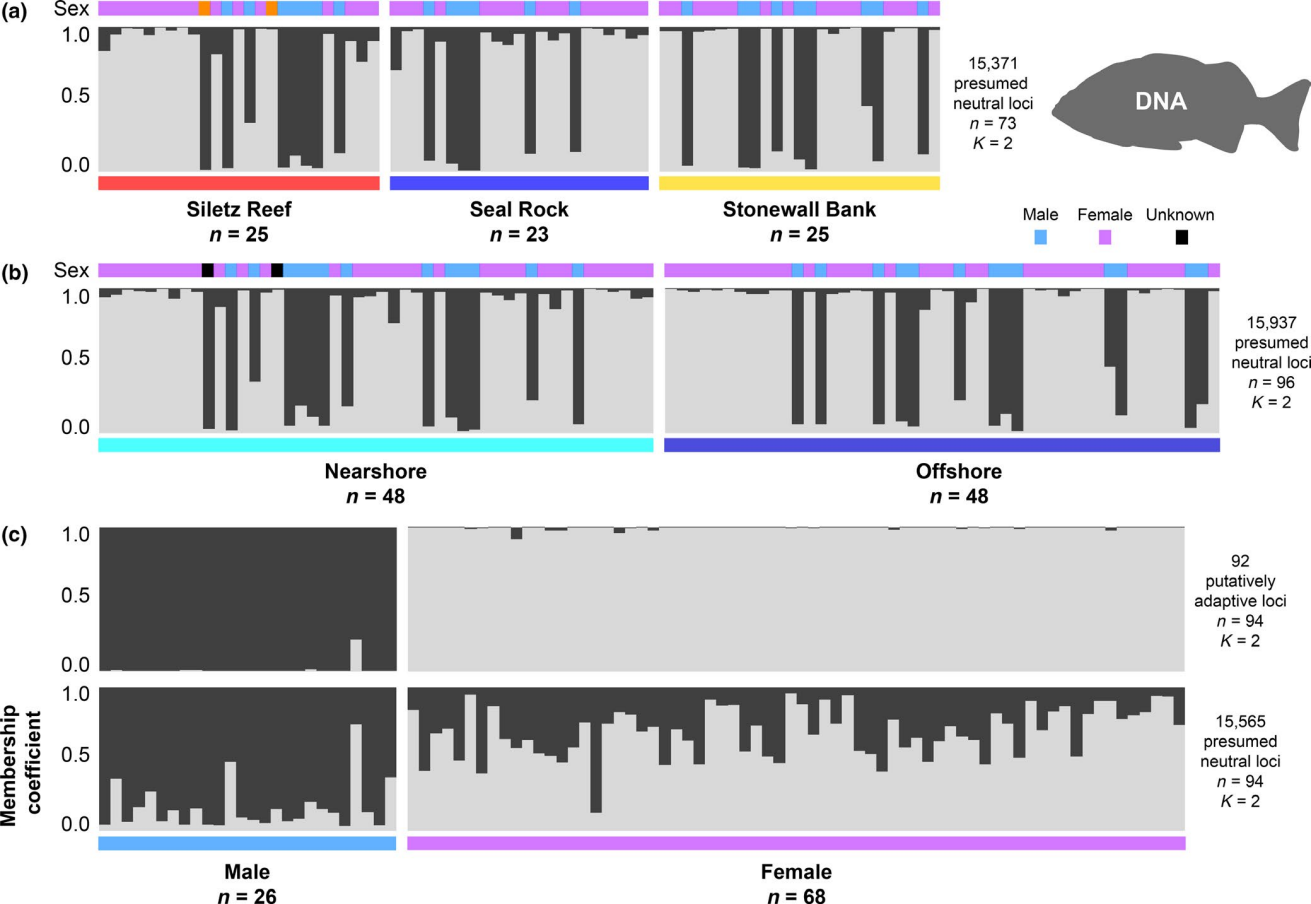
structure bar graphs estimated for genetic variation among deacon rockfish. Each vertical bar represents a separate individual in each dataset and the genotypic clusters estimated among individuals are shown in different colors (light gray and charcoal). The height of a cluster within each vertical bar indicates the confidence that a particular individual is assigned to a given genotypic cluster (referred to as the membership coefficient). The bar graphs show results for each dataset: (a) three sites, (b) two groups, and (c) sex. For sex, putatively adaptive and presumed neutral loci are separated. A colored bar above the graphs denotes the sex of individuals, with males (blue), females (purple), and unknown sex (orange), and bars below the graphs indicate group membership

Genotypic clustering results estimated by structure were similar to the PCA and pairwise *F*_ST_ results for each dataset (Figure 4). The optimal number of clusters for the three sample site and nearshore versus offshore dataset was one or two (Figures S11 and S12), and the two clusters identified for both datasets separated most males and females (Figure 4). In the sex comparison dataset, the optimal number of clusters for the 92 outlier loci was two and the optimal number for the remaining neutral loci was one or two (Figures S11 and S12). The optimal number of clusters for the female‐only and male‐only three sample site datasets was one (Figures S13 and S14). Overall, the structure results indicated that males and females could be distinguished using both neutral and outlier loci, but that none of the sample sites across the remaining datasets could be distinguished, even when organized into nearshore and offshore groups.

### 3.4 Identity of outlier loci

We investigated the genomic identity of the 92 outlier loci detected for the sex comparison dataset. Some of the 92 outlier loci had an allele unique to males, but most loci instead exhibited an allele that was present in all 26 males but rare among females (e.g., 3/68 females). Using BLASTN, most outlier sequences aligned with highest confidence to nuclear DNA sequences of *Scophthalmus maximus* (Linnaeus, 1758) and *Larimichthys crocea* (Richardson, 1846). A subset of outliers aligned with highest confidence to unannotated nuclear DNA sequences of *Sparus aurata* (Linnaeus, 1758), which were previously estimated as quantitative trait loci associated with sex determination and body growth (Loukovitis et al., 2015). Using BLASTN, our outlier loci did not appear to match any of the 26 male‐specific, sex‐linked loci identified in black‐and‐yellow and gopher rockfish by Fowler and Buonaccorsi (2016). Using the BWA‐MEM algorithm, 91 of our 92 outliers successfully mapped to 86 unannotated scaffold sequences of the flag rockfish. Using BLAT, these scaffold sequences were mapped against the annotated genomes of three‐spined stickleback, fugu, and Japanese medaka, with 62 aligning to chromosomes 2 and 3, 13 and 22, and 3 and 17 in each species, respectively.

## 4 DISCUSSION

### 4.1 Concordance between otolith shape and genetic variation

The three sample sites (Siletz Reef, Seal Rock, and Stonewall Bank) could be distinguished using both otolith shape and genetic data when analyzed independently or organized into the nearshore and offshore groups. Although there was substantial overlap, statistically significant differences in otolith shape were found among the sample sites and the tested groups (Table 3, Figure 2). Estimated pseudo *F*‐values for the PERMANOVA test (Table 3) were similar to results reported in previous shaper otolith shape analyses of fish populations sampled over larger geographic distances (Berg et al., 2018; Lee, Brewin, Brickle, & Randhawa, 2018; Libungan, Slotte, Husebø, Godiksen, & Pálsson, 2015; Soeth et al., 2018). Additionally, a similar pattern of otolith shape variation was reported for two rockfish species sampled across the North Atlantic Ocean (Stransky, 2005). This comparability suggests that the differences in otolith shape observed for deacon rockfish are similar to those observed for populations spanning the entire North Atlantic Ocean, suggesting that they may be substantial. Resampling analyses also indicated that sample size was unlikely to have influenced the otolith shape results (Figures S3–S5). Previous otolith shape studies have sampled a large number (≥350) of individuals (e.g., Cañas, Stransky, Schlickeisen, Sampedro, & Fariña, 2012; Stransky, Murta, Schlickeisen, & Zimmermann, 2008b; Stransky, Naumann, et al., 2008a), but many others have sampled ≤100 individuals (e.g., Duncan, Brophy, & Arrizabalaga, 2018; Zhuang et al., 2015), which therefore may have had results affected by sample size effects. The analysis of potential sample size effects indicated that our total otolith sampling (676 specimens) was likely to represent accurate biological variation among the tested groups without significant sample size bias (Figures S3–S5).

We investigated genetic differentiation using pairwise *F*_ST_ and found that the three sample sites and the nearshore and offshore groups were significantly different based on neutral loci (Table 6). However, the estimated genetic difference among potential groups was very low (pairwise *F*_ST_ range of 0.0004–0.013; Table 6). Low *F*_ST_ values are often observed for marine fish (Knutsen et al., 2011; Nielsen et al., 2009), but our results lie within the lower end of the *F*_ST_ range (<0.001–0.07) reported by other RAD sequencing studies of marine fishes (reviewed by Benestan et al., 2017). Similar pairwise *F*_ST_ values (0.0000–0.0276) have been reported by population genetic studies of other rockfish species sampled over larger geographic scales (>2,000 km^2^), and which used the same *SbfI* restriction enzyme (Andrews et al., 2018; Martinez, Buonaccorsi, Hyde, & Aguilar, 2017). Based on the shared RAD sequencing protocol and the results of the power analysis conducted by Martinez et al. (2017), it is likely that our >15,000 loci (Table 5) provided sufficient statistical power to detect fine‐scale population genetic structure, and that our genetic results are accurate.

Despite the statistical significance of our low *F*_ST_ results, the broken‐stick test did not identify any significant axes of variation in the geographic group datasets. There was no obvious geographical structure among samples when these nonsignificant PCs were visualized (Figure 3a,b). No outlier loci were identified in the datasets comparing the three samples sites or nearshore and offshore groups, despite analyzing >15,000 loci (Table 5). Similarly, genotypic clusters estimated by structure analysis did not align with geographic sampling (Figure 4). These results suggest that there are no substantial adaptive genetic differences among these sample sites or between the nearshore and offshore groups. The observed genetic variation based on neutral loci may reflect genetic drift and stochastic demographic changes (i.e., population size and migration rates).

### 4.2 Influence of sex

Sex had an observable effect in our genetic datasets (Figures 3 and 4). This result is consistent with previous reports that have stressed the importance of accounting for sex in reduced representation sequencing studies, where sex‐linked variation can cause erroneous estimates of population genetic differentiation (Benestan et al., 2017; Catchen, 2017). We identified 92 outlier loci associated with sex (Table 5C).

Males and females were significantly different based on both the presumed neutral and outlier loci (Table 6C; Figure 4). Although significant, the *F*_ST_ value based on neutral loci (0.0036) was low and comparable to results for the sample sites, whereas the *F*_ST_ estimate based on the outlier loci was much higher (*F*_ST_ 0.45; Table 6C). Since three of the applied genome scan programs identified *F*_ST_ outliers, the higher *F*_ST_ value estimated for outliers in the sex comparison dataset are expected. Removing 92 outlier loci associated with sex from the datasets comparing the three samples sites and the nearshore and offshore groups did not have a significant effect on pairwise *F*_ST_ results (Table S5). This finding indicates that genetic differentiation among the sample sites was therefore not solely driven by variation for these 92 outlier loci associated with sex.

Pairwise *F*_ST_ estimates and PCA indicated that males and females exhibited a similar pattern of genetic variation among the three sample sites (Table 6D,E; Figure S9). The *F*_ST_ estimate was statistically significant for the Siletz Reef and Stonewall Bank comparison in both the female‐only and male‐only datasets, and for Siletz Reef and Seal Rock comparison in the female‐only dataset (Table 6D,E). However, all other pairwise comparisons were not statistically significant. The lack of genetic differentiation between Seal Rock and Stonewall Bank in both the male and female‐only datasets could be attributed to the shorter geographic distance between these sample sites (24 km) compared to the distance between each site and Siletz Reef (>40 km). On the other hand, the lack of differentiation between Siletz Reef and Seal Rock in the male‐only dataset may be attributed to the small sample size (*n* = 20). In concordance, genotypic cluster analysis in structure also did not identify any population structure among the three sample sites using either sex‐specific dataset. Altogether, these results suggest that genetic differences between male and females are unlikely to have influenced comparisons of the three sample sites.

Potential genetic differences between males and females were not examined in the previous RAD sequencing studies of other rockfish species (Andrews et al., 2018; Martinez et al., 2017). In the study of grass rockfish *Sebastes rastrelliger* (Jordan & Gilbert, 1880), sex was not recorded and PCA plots were not presented (Martinez et al., 2017). However, sex was recorded for canary rockfish and bocaccio *Sebastes paucipinis* (Ayres, 1854), and no obvious geographic structure was observed for either dataset using PCA (Andrews et al., 2018). In yelloweye rockfish, sex was again recorded, but the first two PCs reflected obvious geographic structure among sampling regions (Andrews et al., 2018). Comparison of these findings suggests that the genetic difference estimated between male and female deacon rockfish is relatively strong within the *Sebastes* genus, but the potential cause is unknown until further biological information is available for the species. Overall, our results indicate that variation associated with sex should be explicitly investigated in future population genetic studies of rockfish to avoid potential misinterpretation of data.

### 4.3 Identity of outlier loci

The 92 outlier loci identified between males and females are associated with sex, but do not appear to be strictly sex‐linked, as the outlier loci are variant positions shared between males and females. Some of the outlier loci had an allele exclusive to males, but conversely many loci instead exhibited an allele in all males as well as a small number of females. This bias in genetic variation suggests that there may be differential selection (intralocus sexual conflict) between males and females for autosomal or pseudoautosomal regions (segments of sex chromosomes that recombine) in the deacon rockfish genome. Adaptive genetic differences can occur between the sexes if males and females are placed under different selection pressures for traits such as reproduction and behavior (Bonduriansky & Chenoweth, 2009; Cox & Calsbeek, 2009; Kasimatis, Nelson, & Phillips, 2017). The genetic basis for such traits can be attributed to variation at a single locus (Bonduriansky & Chenoweth, 2009; Bonduriansky, Maklakov, Zajitschek, & Brooks, 2008; Cox & Calsbeek, 2009; Kasimatis et al., 2017; Mank, 2017; Parker & Partridge, 1998; Rowe, Chenoweth, & Agrawal, 2018), which can lead to high estimates of *F*_ST_ (Flanagan & Jones, 2017b; Lucotte, Laurent, Heyer, Ségurel, & Toupance, 2016), as observed in this study (Table 6C). A recent RAD sequencing study of gulf pipefish *Syngnathus scovelli* (Evermann & Kendall, 1896) found that males typically possessed the minor allele, whereas females had the major (Flanagan & Jones, 2017b), which is the same pattern observed for most of the 92 deacon rockfish outlier loci in this study. Since the deacon rockfish was only recently discovered, there is currently insufficient biological information to hypothesize potential drives for selection between males and females, and this subject warrants future investigation.

The sex determination system of deacon rockfish is currently unknown. Previous karyotype and genetic research on other rockfish species have indicated both XY and ZW heterogametic systems within the genus (Anderson, 1979; Fowler & Buonaccorsi, 2016; Ida, Iwasawa, & Kamitori, 1982). An alternative hypothesis for these 92 outlier loci is that they occur on an X chromosome and that male deacon rockfish are the heterogametic sex with XY sex chromosomes. Under this scenario, males would have a single X chromosome copy of these loci that may have been misinterpreted as homozygous sites compared to the same loci in females where there are two X chromosome copies and potential for heterozygosity. If true, we would expect individual males to be consistently homozygous for either allele observed in females. Instead, most loci were heterozygous for members of both sexes, and some loci exhibited an allele exclusive to males or an allele that were frequent for males but rare among females. Given this pattern, it seems more likely that intralocus sexual conflict is the cause. Future investigations should investigate the influence of heterogametic loci when identifying loci and biallelic SNPs in RAD sequencing programs such as stacks.

If the 92 outlier loci occur within a pseudoautosomal region of potential sex chromosomes in deacon rockfish, we could expect most of the outliers to occur within the same genomic region. We tested this hypothesis by modifying the approach of Fowler and Buonaccorsi (2016). All but one of our 92 outlier loci successfully mapped to 86 unannotated scaffold sequences of the flag rockfish, and in turn, 62 of these flag rockfish scaffold sequences aligned to chromosomes 2 and 3, 13 and 22, and 3 and 7 in three‐spined stickleback, fugu, and medaka, respectively. In contrast, most scaffolds associated with the 26 male‐specific, sex‐linked loci for black‐and‐yellow and gopher rockfish, identified by Fowler and Buonaccorsi (2016), mapped instead to chromosomes 14, 12, and 6 in each respective BLAT species. These results suggest that our outlier loci occur on two chromosomes, and not on the equivalent chromosome to the Y chromosome identified in black‐and‐yellow and gopher rockfish (Fowler & Buonaccorsi, 2016).

It is therefore uncertain whether the outliers represent two autosomal regions of the deacon rockfish genome strongly associated with differential selection between the sexes, or if the outliers are pseudoautosomal and deacon rockfish exhibit different sex chromosomes to black‐and‐yellow and gopher rockfish. In addition, the small but significant *F*_ST_ value for the presumed neutral loci between males and females suggests that the sexes may differ for many other positions throughout the genome. Since the karyotypes of other rockfish species have indicated both XY and ZW sex determination systems (Anderson, 1979; Ida et al., 1982), and the last common ancestor of the black‐and‐yellow, gopher, and the blue rockfish is estimated to have occurred ~6.2 Mya (Hyde & Vetter, 2007), it is possible that deacon rockfish have evolved a different set of sex chromosomes or use an alternative sex determination system altogether (e.g., temperature). Intriguingly, a high intensity of intralocus sexual conflict within loci may drive gene duplication and the evolution of new sex determination systems (Gallach & Betrán, 2011; van Doorn, 2009), so the observed 92 outlier loci in deacon rockfish may reflect the ongoing evolution of sex chromosomes in *Sebastes* rockfish (Fowler & Buonaccorsi, 2016). If true, a variable number of outlier loci associated with sex should be observed among rockfish species with different sex determination systems. Differential selection between males and females and change in sex determination systems could also be related to the high level of speciation in *Sebastes* rockfish (Gavrilets, 2014; Parker & Partridge, 1998). A reference genome for a *Sebastes* species should enable future researchers to investigate the function of the 92 sex associated outlier loci for deacon rockfish, as well as potential variance in sex chromosomes. Future validation studies are also required to test the potential adaptive significance of the outlier loci.

### 4.4 Secondary sexual dimorphism in otolith shape

There was a significant difference in male and female otolith shape when all 660 available samples were analyzed together, which was an order of magnitude higher than the results comparing the three sample sites, or the nearshore and offshore groups (Table 3C, Figure 3). Previous geometric morphometric research has found mixed evidence for secondary sexual dimorphism in otolith morphology. Nonsignificant otolith shape differences have been reported for Atlantic herring *Clupea harengus* Linnaeus, 1758, and Atlantic mackerel *Scomber scombrus* Linnaeus, 1758 (Bird et al., 1986; Castonguay et al., 1991). In contrast, although otolith shape variation in Atlantic cod *Gadus morhua* Linneaus, 1758, was mostly influenced by differences in growth rates among populations, a small but significant shape difference was observed between males and females (Campana & Casselman, 1993). A recent study by Parmentier, Boistel, Bahri, Plenevaux, and Schwarnzhans (2018) reported substantial secondary sexual dimorphism in the hearing apparatus and otolith shape of the ophidiid *Neobythites gilli* (Goode and Bean, 1885). Since males and females of this species demonstrated similar hearing ability, it was hypothesized that differences in habitat preference (and associated environmental variables) were responsible for the observed dimorphism (Parmentier et al., 2018).

A potential cause for secondary sexual dimorphism in the otolith shape of deacon Rockfish is uncertain, although otolith shape variation in other rockfish species has been associated with differences in growth rates, habitat usage, and hormone levels (Tuset et al., 2015, 2016). No previous studies have compared otolith morphology and population genetics in rockfish, but Tuset et al. (2016) noted that otolith morphology is more strongly influenced by ecological and biogeographical factors rather than phylogeny. As in other fish lineages (Gauldie & Nelson, 1990; Lombarte & Lleonart, 1993), otolith length and width increases with age in *Sebastes* rockfish, but once fish stop growing in body size, otoliths barely grow in length and instead increase in thickness (Love et al., 2002). Like other rockfish, female deacon rockfish appear to reach larger body sizes than males (Hannah et al., 2015; Love et al., 2002). This difference could cause male otoliths to grow thicker for a greater proportion of their lifespan, generating a relative difference in the shape of male and female otoliths.

A future study with adequate sampling should be able to determine whether age has an influence on the otolith shape difference between males and females. Unfortunately, there is an age bias in our current otolith sampling, with most representatives of the oldest age classes originating from the offshore site of Stonewall Bank (Figure S1). This bias means that we cannot disentangle the sample site variation from the potential influence of age. However, since the sex ratio for the nearshore and offshore groups was similar (1.87 and 1.96, respectively), it is unlikely that the otolith shape difference between these groups was influenced by sex. Ultimately, the linkage of otolith shape to biogeography and ecological variation suggests otolith shape could be a useful tool for stock discrimination in the genus.

### 4.5 Significance of results and implications for fisheries management

Our otolith shape and genetic results indicate a small difference between two potential stocks of deacon rockfish in the nearshore and offshore, which corresponds with the current de facto management for the species. Regardless of whether deacon rockfish were organized into nearshore and offshore groups, morphological and genetic differences were statistically significant but small among the sample sites. Although our morphometric and genetic results were comparable to findings from other marine fishes sampled over larger geographic distances (Benestan et al., 2017; Berg et al., 2018; Lee et al., 2018; Libungan et al., 2015; Soeth et al., 2018), previous stock assessment using similar methods has relied upon stronger patterns in data to delineate a stock boundary (Siegle, Taylor, Miller, Withler, & Yamanaka, 2013; Ward, 2000).

Although differentiation was low, the fact that we detected statistically significant otolith shape and genotypic differences over such a small geographic scale (<50 km^2^) seems remarkable. This statistical significance may reflect the large amount of information (>15,000 loci) provided by the RAD sequencing method. Further genetic sampling across the range of deacon rockfish may help to interpret the scale and significance of variation observed in this study, and improve the distinction of potential stocks. Future genetic studies of rockfish should record the sex of samples and take into account the sex‐ratio and age distribution of sample groups. Inattention to such factors may have already biased the results of previous genetic studies on rockfish (Waples et al., 2008). Future studies of deacon rockfish could investigate the effect of environmental variation on otolith shape across sample sites, as well as differences in habitat usage and feeding preferences. The differences observed for both otolith shape and genetic loci identified in this study may reflect demographic variation within deacon rockfish, which also warrants further investigation. It should be noted that the most recent stock assessment of deacon rockfish did not consider differences in life history parameters between fish from the nearshore and offshore (Dick et al., 2017). However, it should also be remembered that genetic and demographic variation are not always concordant (see discussion by Lowe & Allendorf, 2010; Waples et al., 2008).

Nonetheless, given that we ultimately regard a “fish stock” as a practical management decision (Carvalho & Hauser, 1994; Hilborn & Walters, 1992), it may be prudent to treat fish from the nearshore and offshore as distinct fish stocks until the future genetic, environmental, and demographic research is conducted. Deacon rockfish occurring at depths deeper than 55 m have been effectively not been harvested since 2003, and therefore, fish from the nearshore and offshore areas are essentially managed as separate stocks. Managing the nearshore and offshore fish separately is therefore unlikely to change the current status of the biological populations involved, and this seems to be the most conservative approach until further information is available. We suggest that future assessments and management decisions consider the ramifications of managing this species as one or two stocks. Future research sampling across the entire range of the deacon rockfish, and sequencing DNA from a larger number of individuals, may provide statistical power to differentiate potential nearshore and offshore stocks.

### 4.6 Conclusions

We found small but statistically significant otolith shape and genetic differences among deacon rockfish sampled off the Oregon coast, regardless of whether the three sample sites were analyzed independently or organized into nearshore and offshore groups. We suggest that deacon rockfish from the nearshore and offshore are managed separately until further genetic, environmental, and demographic data are available, requiring no practical change from current management practices.

Sex mattered in our otolith shape and genetic analyses. We found evidence for secondary sexual dimorphism in otolith shape, which may reflect differences in the growth, age, and lifespan of males and females. Males and females were readily distinguished in our genetic data, although this is unlikely to have affected comparisons of the sample sites. Our results concur with previous studies that sex should be considered in population genetic research (Benestan et al., 2017; Catchen, 2017), particularly for *Sebastes* species.

We identified 92 outlier loci that are associated with sex in deacon rockfish. These sites likely reflect differential selection between males and females, which should be investigated in other rockfish species with potentially different sex determination systems. A possible biological cause for this selective difference is uncertain in deacon rockfish, due to the recent discovery of the species. This subject warrants further investigation, as the genetic variation may reflect differences between males and females for habitat usage, which in turn could result in different management requirements.

The data generated in this study can contribute to future, more extensive studies of *Sebastes* rockfish diversity. The sequence data are compatible with reads from previous RADseq studies of other rockfish species that also used the *SbfI* restriction enzyme (Andrews et al., 2018; Martinez et al., 2017). The shaper otolith digitization method (Libungan & Pálsson, 2015) easily allows morphometric datasets from other regions or species to be combined as well. In particular, further research is needed to investigate biological and management requirement differences between deacon and blue rockfish.

This study provides a first step toward the investigation of intraspecific variation in a recently described species, and the results emphasize the potential of RAD sequencing to provide substantial population and sexual genetic information for species that have not been previously studied.

## CONFLICT OF INTEREST

None declared.

## AUTHOR CONTRIBUTIONS

Felix Vaux conducted the genetic analysis and wrote much of the manuscript. Leif K. Rasmuson designed the study, collected samples, conducted the shape analysis, and wrote much of the manuscript. Lisa A. Kautzi processed and photographed the otoliths and contributed to writing in the manuscript. Polly S. Rankin designed the study, collected samples, and contributed to writing in the manuscript. Matthew T.O. Blume designed the study, collected samples, and contributed to writing in the manuscript. Kelly A. Lawrence collected samples, produced Figure 1, and contributed to writing in the manuscript. Sandra Bohn extracted DNA from samples, prepared the sequencing library, and contributed to writing in the manuscript. Kathleen G. O'Malley oversaw the project, particularly the genetic analysis, and wrote much of the manuscript.

## Data Availability

Demultiplexed forward and reverse DNA sequence reads for the deacon rockfish sequenced in this study are openly available on the NCBI sequence read archive (SRA) under: PRJNA560239, http://www.ncbi.nlm.nih.gov/bioproject/560239. Spreadsheets for the otolith and genetic sampling, and genetic data (vcf, genepop, and structure genotype files, and the fasta consensus file for the 92 outlier loci) are available from Dryad: https://doi.org/10.5061/dryad.8kprr4xj0

## References

[ece35763-bib-0001] Abaunza, P. , Murta, A. G. , & Stransky, C. (2013). Sampling for interdisciplinary analysis In Cadrin S. X., Kerr L. A., & Mariani S. (Eds.), Stock identification methods: Applications in fishery science (pp. 477–496). San Diego, CA: Academic Press, Elsevier.

[ece35763-bib-0002] Ahrens, C. W. , Rymer, P. D. , Stow, A. , Bragg, J. , Dillon, S. , Umbers, K. D. L. , & Dudaniec, R. Y. (2018). The search for loci under selection: Trends, biases and progress. Molecular Ecology, 27, 1342–1356. 10.1111/mec.14549 29524276

[ece35763-bib-0004] Allendorf, F. W. , Luikart, G. , & Aitken, S. N. (2013). Conservation and the genetics of populations. Chichester, UK: Wiley‐Blackwell.

[ece35763-bib-0005] Altschul, S. F. , Gish, W. , Miller, W. , Myers, E. W. , & Lipman, D. J. (1990). Basic local alignment search tool. Journal of Molecular Biology, 215, 403–410. 10.1016/S0022-2836(05)80360-2 2231712

[ece35763-bib-0006] Anderson, E. C. (2018). whoa: Where's my heterozygotes? Observations on genotyping accuracy. R package version 0.01. Retrieved from https://zenodo.org/badge/latestdoi/142917310

[ece35763-bib-0007] Anderson, K. L. (1979). A karyological investigation of the systematics of the genus *Sebastes* (subgenus *Pteropodus*). Master's thesis, California State University, San Jose, California.

[ece35763-bib-0008] Anderson, M. J. , & Willis, T. J. (2003). Canonical analysis of principal coordinates: A useful method of constrained ordination for ecology. Ecology, 84, 511–525. 10.1890/0012-9658(2003)084[0511:CAOPCA]2.0.CO;2

[ece35763-bib-0009] Andrews, K. S. , Nichols, K. M. , Elz, A. , Tolimieri, N. , Harvey, C. J. , Pacunski, R. , … Tonnes, D. M. (2018). Cooperative research sheds light on the listing status of threatened and endangered rockfish species. Conservation Genetics, 19, 865–875. 10.1007/s10592-018-1060-0

[ece35763-bib-0010] Begg, G. A. , Friedland, K. D. , & Pearce, J. B. (1999). Stock identification and its role in stock assessment and fisheries management: An overview. Fisheries Research, 43, 1–8. 10.1016/S0165-7836(99)00062-4

[ece35763-bib-0011] Benestan, L. , Moore, J.‐S. , Sutherland, B. J. G. , Le Luyer, J. , Maaroufi, H. , Rougeux, C. , … Bernatchez, L. (2017). Sex matters in massive parallel sequencing: Evidence for biases in genetic parameter estimation and investigation of sex determination systems. Molecular Ecology, 26, 6767–6783. 10.1111/mec.14217 28658525

[ece35763-bib-0012] Berg, F. , Almeland, O. W. , Skadal, J. , Slotte, A. , Andersson, L. , & Folkvord, A. (2018). Genetic factors have a major effect on growth, number of vertebrae and otolith shape in Atlantic herring (*Clupea harengus*). PLoS ONE, 13, e0190995 10.1371/journal.pone.0190995 29324892 PMC5764352

[ece35763-bib-0013] Bernardi, G. , Azzurro, E. , Golani, D. , & Miller, M. R. (2016). Genomic signatures of rapid evolution in the bluespotted cornetfish, a Mediterranean Lessepsian invader. Molecular Ecology, 25, 3384–3396. 10.1111/mec.13682 27162055

[ece35763-bib-0014] Bierne, N. , Welch, J. , Loire, E. , Bonhomme, F. , & David, P. (2011). The coupling hypothesis: Why genome scans may fail to map local adaptation genes. Molecular Ecology, 20, 2044–2072. 10.1111/j.1365-294X.2011.05080.x 21476991

[ece35763-bib-0015] Bird, J. L. , Eppler, D. T. , & Checkley, D. M. Jr (1986). Comparisons of herring otoliths using Fourier series shape analysis. Canadian Journal of Fisheries and Aquatic Sciences, 43, 1228–1234. 10.1139/f86-152

[ece35763-bib-0016] Boehlert, G. W. , & Kappenman, R. F. (1980). Variation with latitude in two species of rockfish (*Sebastes pinniger* and *S. diploproa*) from the northeast Pacific Ocean. Marine Ecology Progress Series, 3, 1–10.

[ece35763-bib-0017] Bonduriansky, R. , & Chenoweth, S. F. (2009). Intralocus sexual conflict. Trends in Ecology and Evolution, 24, 280–288. 10.1016/j.tree.2008.12.005 19307043

[ece35763-bib-0018] Bonduriansky, R. , Maklakov, A. , Zajitschek, F. , & Brooks, R. (2008). Sexual selection, sexual conflict and the evolution of ageing and life span. Functional Ecology, 22, 443–453. 10.1111/j.1365-2435.2008.01417.x

[ece35763-bib-0019] Bouaziz, M. , Jeanmougin, M. , & Geudj, M. (2012). Chapter 13: Multiple testing in large‐scale genetic studies In Pompanon F., & Bonin A. (Eds.), Data production and analysis in population genomics: Methods and protocols, methods in molecular biology 888 (pp. 213–233). New York, NY: Springer Science + Business Media. 10.1007/978-1-61779-870-2_13 22665284

[ece35763-bib-0020] Burford, M. O. (2009). Demographic history, geographical distribution and reproductive isolation of distinct lineages of blue rockfish (*Sebastes mystinus*) a marine fish with high dispersal potential. Journal of Evolutionary Biology, 22, 1471–1486. 10.1111/j.1420-9101.2009.01760.x 19467131

[ece35763-bib-0021] Burford, M. O. , & Bernardi, G. (2008). Incipient speciation within a subgenus of rockfish (*Sebastosomus*) provides evidence of recent radiations within an ancient species flock. Marine Biology, 154, 701–717. 10.1007/s00227-008-0963-6

[ece35763-bib-0022] Burford, M. O. , Carr, M. H. , & Bernardi, G. (2011). Age‐structure genetic analysis reveals temporal and geographic variation within and between two cryptic rockfish species. Marine Ecology Progress Series, 442, 201–215. 10.3354/meps09329

[ece35763-bib-0023] Cadrin, S. X. , Karr, L. A. , & Mariani, S. (2013). Stock identification methods: An overview In Cadrin S. X., Kerr L. A., & Mariani S. (Eds.), Identification methods: Applications in fishery science (pp. 1–5). San Diego, CA: Academic Press, Elsevier.

[ece35763-bib-0024] Cadrin, S. X. , & Secor, D. H. (2009). Accounting for spatial population structure in stock assessment: Past, present and future In Beamish R. J., & Rothschild B. J. (Eds.), The future of fisheries science in North America, fish & fisheries series (pp. 405–426). Berlin, Germany: Springer Science + Business Media B.V.

[ece35763-bib-0025] Campana, S. E. , & Casselman, J. M. (1993). Stock discrimination using otolith shape analysis. Canadian Journal of Fisheries and Aquatic Sciences, 50, 1062–1083. 10.1139/f93-123

[ece35763-bib-0026] Cañas, L. , Stransky, C. , Schlickeisen, J. , Sampedro, M. P. , & Fariña, A. C. (2012). Use of otolith shape analysis in the identification of anglerfish (*Lophius piscatorius*) in the northeast Atlantic. ICES Journal of Marine Science, 69, 250–256. 10.1093/icesjms/fss006

[ece35763-bib-0027] Cangelosi, R. , & Goriely, A. (2007). Component retention in principal component analysis with application to cDNA microarray data. Biology Direct, 2, 2 10.1186/1745-6150-2-2 17229320 PMC1797006

[ece35763-bib-0028] Carvalho, G. R. , & Hauser, L. (1994). Molecular genetics and the stock concept in fisheries. Reviews in Fish Biology and Fisheries, 4, 326–350. 10.1007/BF00042908

[ece35763-bib-0029] Castonguay, M. , Simard, P. , & Gagnon, P. (1991). Usefulness of Fourier analysis of otolith shape for Atlantic mackerel (*Scomber scombrus*) stock discrimination. Canadian Journal of Fisheries and Aquatic Sciences, 48, 296–302. 10.1139/f91-041

[ece35763-bib-0030] Catchen, J. M. (2017). When structure leads to sex: Untangling signals in population genetic data sets. Molecular Ecology, 26, 6763–6766. 10.1111/mec.14422 29282805

[ece35763-bib-0031] Catchen, J. M. , Amores, A. , Hohenlohe, P. , & Postlethwait, J. H. (2011). Stacks: Building and genotyping loci de novo from short‐read sequences. G3: Genes, Genomics, Genetics, 1, 171–182. 10.1534/g3.111.000240 PMC3276136 22384329

[ece35763-bib-0032] Catchen, J. M. , Hohenlohe, P. A. , Bassham, S. , Amores, A. , & Cresko, W. A. (2013). Stacks: An analysis tool set for population genomics. Molecular Ecology, 22, 3124–3140. 10.1111/mec.12354 23701397 PMC3936987

[ece35763-bib-0033] Chilton, D. E. , & Beamish, R. J. (1982). Age determination methods for fishes studied by the ground fish program of the Pacific Biological Station. Canadian Special Publication of Fisheries and Aquatic Sciences, 60, 102.

[ece35763-bib-0034] Christensen, H. T. , Rigét, F. , Backe, M. B. , Saha, A. , Johansen, T. , & Hedeholm, R. B. (2018). Comparison of three methods for identification of redfish (*Sebastes mentella* and *S. norvegicus*) from the Greenland east coast. Fisheries Research, 201, 11–17. 10.1016/j.fishres.2018.01.003

[ece35763-bib-0035] Cope, J. M. (2004). Population genetics and phylogeography of the blue rockfish (*Sebastes mystinus*) from Washington to California. Canadian Journal of Fisheries and Aquatic Sciences, 61, 332–342. 10.1139/f04-008

[ece35763-bib-0036] Cox, R. M. , & Calsbeek, R. (2009). Sexually antagonistic selection, sexual dimorphism, and the resolution of intralocus sexual conflict. American Naturalist, 173, 176–187. 10.1086/595841 19138156

[ece35763-bib-0037] Coyle, T. (1998). Stock identification and fisheries management: The importance of using several methods in a stock identification study In Hancock D. A. (Ed.), Taking stock: Defining and managing shared resources (pp. 173–182). Sydney, Australia: Australian Society for Fishery Biology.

[ece35763-bib-0038] Danecek, P. , Auton, A. , Abecasis, G. , Albers, C. A. , Banks, E. , DePristo, M. A. , … 1000 Genomes Project Analysis Group (2011). The variant call format and VCFtools. Bioinformatics, 27, 2156–2158. 10.1093/bioinformatics/btr330 21653522 PMC3137218

[ece35763-bib-0039] Dick, A. J. , Berger, A. , Bizzarro, J. , Bosley, K. , Cope, J. , Field, J. , … … (2017). The combined status of blue and deacon rockfishes in the U.S. waters off California and Oregon in 2017. Portland, OR: Pacific Fishery Management Council.

[ece35763-bib-0040] Duncan, R. , Brophy, D. , & Arrizabalaga, H. (2018). Otolith shape analysis as a tool for stock separation of albacore tuna feeding in the northeast Atlantic. Fisheries Research, 200, 68–74. 10.1016/j.fishres.2017.12.011

[ece35763-bib-0041] Earl, D. A. , & vonHoldt, B. M. (2012). STRUCTURE HARVESTER: A website and program for visualising STRUCTURE output and implementing the Evanno method. Conservation Genetics Resources, 4, 359–361. 10.1007/s12686-011-9548-7

[ece35763-bib-0042] Evanno, G. , Regnaut, S. , & Goudet, J. (2005). Detecting the number of clusters of individuals using the software STRUCTURE: A simulation study. Molecular Ecology, 14, 2611–2620. 10.1111/j.1365-294X.2005.02553.x 15969739

[ece35763-bib-0043] Excoffier, L. , & Ray, N. (2008). Surfing during population expansions promotes genetic revolutions and structuration. Trends in Ecology and Evolution, 23, 347–351. 10.1016/j.tree.2008.04.004 18502536

[ece35763-bib-0044] Flanagan, S. P. , & Jones, A. G. (2017a). Constraints on the *F* _ST_‐heterozygosity outlier approach. Journal of Heredity, 108, 561–573. 10.1093/jhered/esx048 28486592

[ece35763-bib-0045] Flanagan, S. P. , & Jones, A. G. (2017b). Genome‐wide selection components analysis in a fish with male pregnancy. Evolution, 71, 1096–1105. 10.1111/evo.13173 28067418

[ece35763-bib-0046] Foll, M. , & Gaggiotti, O. (2008). A genome‐scan method to identify selected loci appropriate for both dominant and codominant markers: A Bayesian perspective. Genetics, 180, 977–993. 10.1534/genetics.108.092221 18780740 PMC2567396

[ece35763-bib-0047] Fowler, B. L. S. , & Buonaccorsi, V. P. (2016). Genomic characterization of sex‐identification markers in *Sebastes carnatus* and *Sebastes chrysomelas* rockfishes. Molecular Ecology, 25, 2165–2175. 10.1111/mec.13594 26923740

[ece35763-bib-0048] Frable, B. W. , Wagman, D. W. , Frierson, T. N. , Aguilar, A. , & Sidlauskas, B. L. (2015). A new species of *Sebastes* (Scorpaeniformes: Sebastidae) from the northeastern Pacific, with a redescription of the blue rockfish, *S. mystinus* (Jordan and Gilbert, 1881). Fisheries Bulletin, 113, 355–377. 10.7755/FB.113.4.1

[ece35763-bib-0049] Frey, P. H. , Head, M. A. , & Keller, A. A. (2015). Maturity and growth of darkblotched rockfish, *Sebastes crameri*, along the U.S. west coast. Environmental Biology of Fishes, 98, 2353–2365. 10.1007/s10641-015-0441-1

[ece35763-bib-0050] Funk, W. C. , McKay, J. K. , Hohenlohe, P. A. , & Allendorf, F. W. (2012). Harnessing genomics for delineating conservation units. Trends in Ecology and Evolution, 27, 489–496. 10.1016/j.tree.2012.05.012 22727017 PMC4185076

[ece35763-bib-0051] Gagnaire, P.‐A. , Broquet, T. , Aurelle, D. , Viard, F. , Souissi, A. , Bonhomme, F. , … Bierne, N. (2015). Using neutral, selected, and hitchhiker loci to assess connectivity of marine populations in the genomic era. Evolutionary Applications, 8, 769–786. 10.1111/eva.12288 26366195 PMC4561567

[ece35763-bib-0052] Gallach, M. , & Betrán, E. (2011). Intralocus sexual conflict resolved through gene duplication. Trends in Ecology and Evolution, 26, 222–228. 10.1016/j.tree.2011.02.004 21397976 PMC3090214

[ece35763-bib-0053] Gauldie, R. W. , & Nelson, D. G. A. (1990). Otolith growth in fishes. Comparative Biochemistry and Physiology A, 97, 119–135. 10.1016/0300-9629(90)90159-P

[ece35763-bib-0054] Gavrilets, S. (2014). Is sexual conflict an “engine of speciation”? Cold Spring Harbor Perspectives in Biology, 11, a917723 10.1101/cshperspect.a017723 PMC4292158 25395295

[ece35763-bib-0055] Gertseva, V. V. , Cope, J. M. , & Matson, S. E. (2010). Growth variability in the splitnose rockfish *Sebastes diploproa* of the northeast Pacific Ocean: Pattern revisited. Marine Ecology Progress Series, 413, 125–136. 10.3354/meps08719

[ece35763-bib-0056] Grummer, J. A. , Beheregaray, L. B. , Bernatchez, L. , Hand, B. K. , Luikart, G. , Narum, S. R. , & Taylor, E. B. (2019). Aquatic landscape genomics and the environmental effects on genetic variation. Trends in Ecology and Evolution, 34, 641–654. 10.1016/j.tree.2019.02.013 30904190

[ece35763-bib-0057] Hannah, R. E. , Buell, T. V. , & Blume, M. T. O. (2008). Reducing bycatch in Oregon's recreational groundfish fishery: Experimental results with angling gear configured to increase bait height above bottom. Information reports 2008‐03. Newport, OR: Oregon Department of Fish and Wildlife.

[ece35763-bib-0058] Hannah, R. W. , Wagman, D. W. , & Kautzi, L. A. (2015). Cryptic speciation in the blue rockfish (*Sebastes mystinus*): Age, growth and female maturity of the blue‐sided rockfish, a newly identified species, from Oregon waters. Information reports 2015‐01. Newport, OR: Oregon Department of Fish and Wildlife.

[ece35763-bib-0059] Hauser, L. , & Carvalho, G. R. (2008). Paradigm shifts in marine fisheries genetics: Ugly hypotheses slain by beautiful facts. Fish and Fisheries, 9, 333–362. 10.1111/j.1467-2979.2008.00299.x

[ece35763-bib-0060] Hilborn, R. , & Walters, C. J. (1992). Quantitative fisheries stock assessment. Choice, dynamics and uncertainty. London, UK: Chapman and Hall.

[ece35763-bib-0061] Hyde, J. R. , & Vetter, R. D. (2007). The origin, evolution, and diversification of rockfishes of the genus *Sebastes* (Cuvier). Molecular Phylogenetics and Evolution, 44, 790–811. 10.1016/j.ympev.2006.12.026 17320419

[ece35763-bib-0062] Ida, H. , Iwasawa, T. , & Kamitori, M. (1982). Karyotypes in 8 species of *Sebastes* from Japan. Japanese Journal of Ichthyology, 29, 162–168.

[ece35763-bib-0063] Ivanova, N. V. , Dewaard, J. R. , & Hebert, P. D. N. (2006). An inexpensive, automation‐friendly protocol for recovering high‐quality DNA. Molecular Ecology Resources, 6, 988–1002. 10.1111/j.1471-8286.2006.01428.x

[ece35763-bib-0064] Jackson, D. A. (1993). Stopping rules in principal components analysis: A comparison of heuristical and statistical approaches. Ecology, 74, 2204–2214. 10.2307/1939574

[ece35763-bib-0065] Jombart, T. (2008). adegenet: A R package for the multivariate analysis of genetic markers. Bioinformatics, 24, 1403–1405. 10.1093/bioinformatics/btn129 18397895

[ece35763-bib-0066] Jombart, T. , & Ahmed, I. (2011). adegenet 1.3‐1: New tools for the analysis of genome‐wide SNP data. Bioinformatics, 27, 3070–3071. 10.1093/bioinformatics/btr521 21926124 PMC3198581

[ece35763-bib-0067] Kasimatis, K. R. , Nelson, T. C. , & Phillips, P. C. (2017). Genomic signatures of sexual conflict. Journal of Heredity, 108, 780–790. 10.1093/jhered/esx080 29036624 PMC5892400

[ece35763-bib-0068] Kent, J. W. (2002). BLAT – The BLAST‐like alignment tool. Genome Research, 12, 656–664. 10.1101/gr.229202 11932250 PMC187518

[ece35763-bib-0069] Knutsen, H. , Olsen, E. M. , Jorde, P. E. , Espeland, S. H. , André, C. , & Stenseth, N. C. (2011). Are low but statistically significant levels of genetic differentiation in marine fishes ‘biologically meaningful’? A case study of coastal Atlantic cod. Molecular Ecology, 20, 768–783. 10.1111/j.1365-294X.2010.04979.x 21199035

[ece35763-bib-0070] Kumar, G. , & Kocour, M. (2017). Applications of next‐generation sequencing in fisheries research: A review. Fisheries Research, 186, 11–22. 10.1016/j.fishres.2016.07.021

[ece35763-bib-0071] Lea, E. (1929). The oceanic stage in the life history of Norwegian herring. ICES Journal of Marine Science, 4, 3–42. 10.1093/icesjms/4.1.3

[ece35763-bib-0072] Lee, B. , Brewin, P. E. , Brickle, P. , & Randhawa, H. (2018). Use of otolith shape to inform stock structure in Patagonian toothfish (*Dissostichus eleginoides*) in the south‐western Atlantic. Marine and Freshwater Research, 69, 1238–1247. 10.1071/MF17327

[ece35763-bib-0073] Li, H. , & Durbin, R. (2009). Fast and accurate short read alignment with Burrows‐Wheeler transform. Bioinformatics, 25, 1754–1760. 10.1093/bioinformatics/btp324 19451168 PMC2705234

[ece35763-bib-0074] Li, Z. , Gray, A. K. , Love, M. S. , Asahida, T. , & Gharrett, A. J. (2006). Phylogeny of members of the rockfish (*Sebastes*) subgenus *Pteropodus* and their relatives. Canadian Journal of Zoology, 84, 527–536. 10.1139/z06-022

[ece35763-bib-0075] Libungan, L. A. , & Pálsson, S. (2015). ShapeR: An R package to study otolith shape variation among fish populations. PLoS ONE, 10, e0121102 10.1371/journal.pone.0121102 25803855 PMC4372608

[ece35763-bib-0076] Libungan, L. A. , Slotte, A. , Husebø, Å. , Godiksen, J. A. , & Pálsson, S. (2015). Latitudinal gradient in otolith shape among local populations of Atlantic herring (*Clupea harengus* L.) in Norway. PLoS ONE, 10, e01300847 10.1371/journal.pone.0130847 PMC4478005 26101885

[ece35763-bib-0077] Lischer, H. E. , & Excoffier, L. (2012). PGDSpider: An automated data conversion tool for connecting population genetics and genome programs. Bioinformatics, 28, 298–299. 10.1093/bioinformatics/btr642 22110245

[ece35763-bib-0078] Lleonart, J. , Salat, J. , & Torres, G. J. (2000). Removing allometric effects of body size in morphological analysis. Journal of Theoretical Biology, 205, 85–93. 10.1006/jtbi.2000.2043 10860702

[ece35763-bib-0079] Lombarte, A. , & Lleonart, J. (1993). Otolith size changes related with body growth, habitat depth and temperature. Environmental Biology of Fishes, 37, 297–306. 10.1007/BF00004637

[ece35763-bib-0080] Loukovitis, D. , Sarrapoulou, E. , Tsigenopolos, C. S. , Batargias, C. , Magoulas, A. , Apostolidis, A. P. , … Kotoulas, G. (2015). Quantitative trait loci involved in sex determination and body growth in the gilthead sea bream (*Sparus aurata* L.) through targeted genome scan. PLoS ONE, 6, e16599 10.1371/journal.pone.0016599 PMC3031595 21304996

[ece35763-bib-0081] Love, M. S. , Yoklavich, M. , & Thorsteinson, L. (2002). The Rockfishes of the Northeast Pacific. Berkeley, LA: University of California Press.

[ece35763-bib-0082] Lowe, W. H. , & Allendorf, F. W. (2010). What can genetics tell us about population connectivity? Molecular Ecology, 19, 3038–3051. 10.1111/j.1365-294X.2010.04688.x 20618697

[ece35763-bib-0083] Lucotte, E. A. , Laurent, R. , Heyer, E. , Ségurel, L. , & Toupance, B. (2016). Detection of allelic frequency differences between sexes in humans: A signature of sexually antagonistic selection. Genome Biology and Evolution, 8, 1489–1500. 10.1093/gbe/evw090 27189992 PMC4898804

[ece35763-bib-0084] Luu, K. , Bazin, E. , & Blum, M. G. B. (2017). pcadapt: An R package to perform genome scans for selection based on principal component analysis. Molecular Ecology Resources, 17, 67–77. 10.1111/1755-0998.12592 27601374

[ece35763-bib-0085] MacLean, J. A. , & Evans, D. O. (1981). The stock concept, discreteness of fish stocks, and fisheries management. Canadian Journal of Fisheries and Aquatic Sciences, 38, 1889–1898. 10.1139/f81-235

[ece35763-bib-0086] Mank, J. E. (2017). Population genetics of sexual conflict in the genomic era. Nature Reviews, 18, 721–730. 10.1038/nrg.2017.83 29062057

[ece35763-bib-0087] Martinez, E. , Buonaccorsi, V. , Hyde, J. R. , & Aguilar, A. (2017). Population genomics reveals high gene flow in grass rockfish (*Sebastes rastrelliger*). Marine Genomics, 33, 57–63. 10.1016/j.margen.2017.01.004 28169128

[ece35763-bib-0088] Mastretta‐Yanes, A. , Arrigo, N. , Alvarez, N. , Jorgensen, T. H. , Piñeros, D. , & Emerson, B. C. (2015). Restriction site‐associated DNA sequencing, genotyping error estimation and de novo assembly optimization for population genetic inference. Molecular Ecology Resources, 15, 28–41. 10.1111/1755-0998.12291 24916682

[ece35763-bib-0089] McKinney, G. J. , Waples, R. K. , Seeb, L. W. , & Seeb, J. E. (2017). Paralogs are revealed by proportion of heterozygotes and deviations in read ratios in genotyping‐by‐sequencing data from natural populations. Molecular Ecology Resources, 17, 656–669. 10.1111/1755-0998.12613 27762098

[ece35763-bib-0090] Milano, I. , Babbucci, M. , Cariani, A. , Antanassova, M. , Bekkevold, D. , Carvalho, G. R. , … Bargelloni, L. (2014). Outlier SNP markers reveal fine‐scale genetic structuring across European hake populations (*Merluccius merluccius*). Molecular Ecology, 23, 118–135. 10.1111/mec.12568 24138219

[ece35763-bib-0091] Mortiz, E. M. (2018). paralog‐finder v1.0, a script to detect and blacklist paralog RAD loci. 10.5281/zenodo.1209328. Retrieved from https://github.com/edgardomortiz/paralog-finder

[ece35763-bib-0092] Narum, S. R. , & Hess, J. E. (2011). Comparison of *F* _ST_ outlier tests for SNP loci under selection. Molecular Ecology Resources, 11, 184–194. 10.1111/j.1755-0998.2011.02987.x 21429174

[ece35763-bib-0093] Nielsen, E. E. , Hemmer‐Hansen, J. , Foged Larsen, P. , & Bekkevold, D. (2009). Population genomics of marine fishes: Identifying adaptive variation in space and time. Molecular Ecology, 18, 3128–3150. 10.1111/j.1365-294X.2009.04272.x 19627488

[ece35763-bib-0094] Oksanen, J. , Blanchet, F. G. , Kindt, R. , Legendre, P. , Minchin, P. R. , O'Hara, … Wagner, H. (2018). vegan: Community ecology package. R package version 2.5‐2. Retrieved from https://CRAN.R-project.org/package=vegan

[ece35763-bib-0095] Ovenden, J. R. , Berry, O. , Welch, D. J. , Buckworth, R. C. , & Dichmont, C. M. (2015). Ocean's eleven: A critical evaluation of the role of population, evolutionary and molecular genetics in the management of wild fish. Fish and Fisheries, 16, 125–159. 10.1111/faf.12052

[ece35763-bib-0096] Parker, G. A. , & Partridge, L. (1998). Sexual conflict and speciation. Philosophical Transactions of the Royal Society of London. Series B: Biological Sciences, 353, 261–274. 10.1098/rstb.1998.0208 9533125 PMC1692203

[ece35763-bib-0097] Parmentier, E. , Boistel, R. , Bahri, M. A. , Plenevaux, A. , & Schwarnzhans, W. (2018). Sexual dimorphism in the sonic system and otolith morphology of *Neobythites gilli* (Ophidiiformes). Journal of Zoology, 306, 274–280. 10.1111/jzo.12561

[ece35763-bib-0098] Pembleton, L. W. , Cogan, N. O. I. , & Forster, J. W. (2013). StAMPP: An R package for calculation of genetic differentiation and structure of mixed‐ploidy level populations. Molecular Ecology Resources, 13, 946–952. 10.1111/1755-0998.12129 23738873

[ece35763-bib-0099] PFMC (Pacific Fishery Management Council) (2016). Pacific Coast Groundfish Fishery Management Plan for the California, Oregon, and Washington Groundfish Fishery. Silver Spring, MD: National Oceanographic and Atmospheric Administration.

[ece35763-bib-0100] Pritchard, J. K. , Stephens, M. , & Donnelly, P. (2000). Inference of population structure using multilocus genotype data. Genetics, 155, 945–959. 10835412 10.1093/genetics/155.2.945 PMC1461096

[ece35763-bib-0101] Purcell, S. , Neale, B. , Todd‐Brown, K. , Thomas, L. , Ferreira, M. A. R. , Bender, D. , … Sham, P. C. (2007). PLINK: A tool set for whole‐genome association and population‐based linkage analyses. American Journal of Human Genetics, 81, 559–575. 10.1086/519795 17701901 PMC1950838

[ece35763-bib-0102] R Core Team (2018). R: A language environment for statistical computing. Vienna, Austria: R Foundation for Statistical Computing Retrieved from http://www.R-project.org

[ece35763-bib-0103] Ralston, S. (1990). Size selection of snappers (*Lutjanidae*) by hook and line gear. Canadian Journal of Fisheries and Aquatic Sciences, 47, 696–700. 10.1139/f90-078

[ece35763-bib-0104] Research Group, LLC (2015a). Oregon marine recreational fisheries economic contributions in 2013 and 2014. Prepared for Oregon Department of Fish and Wildlife and Oregon Coastal Zone Management Association, Corvallis, Oregon. September 2015.

[ece35763-bib-0105] Research Group, LLC (2015b). Oregon's commercial fishing industry, year 2013 and 2014 review. Prepared for Oregon Department of Fish and Wildlife, and Oregon Coastal Zone Management Association, Corvallis, Oregon. September 2015.

[ece35763-bib-0106] Riginos, C. , Crandall, E. D. , Liggins, L. , Bongaerts, P. , & Treml, E. A. (2016). Navigating the currents of seascape genomics: How spatial analyses can augment population genomic studies. Current Zoology, 62, 581–601. 10.1093/cz/zow067 29491947 PMC5804261

[ece35763-bib-0107] Rowe, L. , Chenoweth, S. F. , & Agrawal, A. F. (2018). The genomics of sexual conflict. American Naturalist, 192, 274–286. 10.1086/698198 30016158

[ece35763-bib-0108] Schindelin, J. , Arganda‐Carreras, I. , Frise, E. , Kaynig, V. , Longair, M. , Pietzsch, T. , … Cardona, A. (2012). Fiji: An open‐source platform for biological‐image analysis. Nature Methods, 9, 676–682. 10.1038/nmeth.2019 22743772 PMC3855844

[ece35763-bib-0109] Shafer, A. B. A. , Wolf, J. B. W. , Alves, P. C. , Bergström, L. , Bruford, M. W. , Brännström, I. , … Zieliński, P. (2015). Genomics and the challenging translation into conservation practice. Trends in Ecology and Evolution, 30, 78–87. 10.1016/j.tree.2014.11.009 25534246

[ece35763-bib-0110] Siegle, M. R. , Taylor, E. B. , Miller, K. M. , Withler, R. E. , & Yamanaka, K. L. (2013). Subtle population genetic structure in yelloweye rockfish (*Sebastes ruberrimus*) is consistent with a major oceanographic division in British Columbia, Canada. PLoS ONE, 8, e71083 10.1371/journal.pone.0071083 23990926 PMC3749191

[ece35763-bib-0111] Sivasundar, A. , & Palumbi, S. R. (2010). Life history, ecology and the biogeography of strong genetic breaks among 15 species of Pacific rockfishes, *Sebastes* . Marine Biology, 157, 1433–1452. 10.1007/s00227-010-1419-3

[ece35763-bib-0112] Smith, B. J. (2007). boa: An R package for MCMC output convergence assessment and posterior inference. Journal of Statistical Software, 21, 1–37. 10.18637/jss.v021.i11

[ece35763-bib-0113] Soeth, M. , Spach, H. L. , Daros, F. A. , Adelier‐Alves, J. , de Almeida, A. C. O. , & Correia, A. T. (2018). Stock structure of Atlantic spadefish *Chaetodipterus faber* from Southwest Atlantic Ocean inferred from otolith elemental and shape signatures. Fisheries Research, 211, 81–90. 10.1016/j.fishres.2018.11.003

[ece35763-bib-0114] Storey, J. D. (2002). A direct approach to false discovery rates. Journal of the Royal Statistical Society: Series B (Statistical Methodology), 64, 479–498. 10.1111/1467-9868.00346

[ece35763-bib-0115] Storey, J. D. , Bass, A. J. , Dabney, A. , & Robinson, D. (2018). qvalue: Q‐value estimation for false discovery rate control. R package version 2.12.0. Retrieved from http://github.com/jdstorey/qvalue

[ece35763-bib-0116] Stransky, C. (2005). Geographic variation of golden redfish (*Sebastes marinus*) and deep‐sea redfish (*S. mentella*) in the North Atlantic based on otolith shape analysis. ICES Journal of Marine Science, 62, 1691–1698. 10.1016/j.icesjms.2005.05.012

[ece35763-bib-0117] Stransky, C. , & MacLellan, S. E. (2005). Species separation and zoogeography of redfish and rockfish (genus *Sebastes*) by otolith shape analysis. Canadian Journal of Fisheries and Aquatic Sciences, 62, 2265–2276. 10.1139/f05-143

[ece35763-bib-0118] Stransky, C. , Murta, A. G. , Schlickeisen, J. , & Zimmermann, C. (2008b). Otolith shape analysis as a tool for stock separation of horse mackerel (*Trachurus trachurus*) in the northeast Atlantic and Mediterranean. Fisheries Research, 89, 159–166. 10.1016/j.fishres.2007.09.017

[ece35763-bib-0119] Stransky, C. , Naumann, H. , Fevolden, S.‐E. , Harbitz, A. , Høie, H. , Nedreaas, K. H. , … Skarsteinm, T. H. (2008a). Separation of Norwegian coastal cod and northeast Arctic cod by outer otolith shape analysis. Fisheries Research, 90, 26–35. 10.1016/j.fishres.2007.09.009

[ece35763-bib-0120] Tuset, V. M. , Imondi, R. , Aguado, G. , Otero‐Ferrer, J. L. , Santschi, L. , Lombarte, A. , & Love, M. (2015). Otolith patterns of rockfishes from the northeastern Pacific. Journal of Morphology, 276, 458–469. 10.1002/jmor.20353 25503537

[ece35763-bib-0121] Tuset, V. M. , Otero‐Ferrer, J. L. , Gomez‐Zurity, J. , Venerus, L. A. , Stransky, C. , Imondi, R. , … Lombarte, A. (2016). Otolith shape lends support to the sensory drive hypothesis in rockfishes. Journal of Evolutionary Biology, 29, 2083–2097. 10.1111/jeb.12932 27364643

[ece35763-bib-0122] Valenzuela‐Quiñonez, F. (2016). How fisheries management can benefit from genomics? Briefings in Functional Genomics, 15, 352–357. 10.1093/bfgp/elw006 26995687

[ece35763-bib-0123] Van Doorn, G. S. (2009). Intralocus sexual conflict. Annals of the New York Academy of Sciences, 1168, 52–71. 10.1111/j.1749-6632.2009.04573.x 19566703

[ece35763-bib-0124] Vignon, M. (2015). Disentangling and quantifying sources of otolith shape variation across multiple scales using a new hierarchical partitioning approach. Marine Ecology Progress Series, 534, 163–177. 10.3354/meps11376

[ece35763-bib-0125] Waldman, J. R. (1999). The importance of comparative studies in stock analysis. Fisheries Research, 43, 237–246. 10.1016/S0165-7836(99)00075-2

[ece35763-bib-0126] Wang, J. (2011). Coancestry: A program for simulating, estimating and analysing relatedness and inbreeding coefficients. Molecular Ecology Resources, 11, 141–145. 10.1111/j.1755-0998.2010.02885.x 21429111

[ece35763-bib-0127] Wang, J. (2017). Estimating pairwise relatedness in a small sample of individuals. Heredity, 119, 302–313. 10.1038/hdy.2017.52 28853716 PMC5637371

[ece35763-bib-0128] Waples, R. S. (2015). Testing for Hardy‐Weinberg proportions: Have we lost the plot? Journal of Heredity, 106, 1–19. 10.1093/jhered/esu062 25425676

[ece35763-bib-0129] Waples, R. S. , Punt, A. E. , & Cope, J. M. (2008). Integrating genetic data into management of marine resources: How can we do it better? Fish and Fisheries, 9, 423–449. 10.1111/j.1467-2979.2008.00303.x

[ece35763-bib-0130] Ward, R. D. (2000). Genetics in fisheries management. Hydrobiologia, 420, 191–201. 10.1023/A:1003928327503

[ece35763-bib-0131] Weir, B. S. , & Cockerham, C. C. (1984). Estimating F‐statistics for the analysis of population structure. Evolution, 38, 1358–1370. 10.1111/j.1558-5646.1984.tb05657.x 28563791

[ece35763-bib-0132] Westgaard, J.‐I. , Saha, A. , Kent, M. , Hansen, H. H. , Knutsen, H. , Hauser, L. , … Johansen, T. (2017). Genetic population structure in Greenland halibut (*Reinhardtius hippoglossoides*) and its relevance to fishery management. Canadian Journal of Fisheries and Aquatic Sciences, 74, 475–485. 10.1139/cjfas-2015-0430

[ece35763-bib-0133] Whitlock, M. C. , & Lotterhos, K. E. (2015). Reliable detection of loci responsible for local adaptation: Inference of a null model through trimming of the distribution of *F* _ST_*. American Naturalist, 186, S24–S36. 10.1086/682949 26656214

[ece35763-bib-0134] Wigginton, J. E. , Cutler, D. J. , & Abecasis, G. (2005). A note on exact tests of Hardy‐Weinberg equilibrium. American Journal of Human Genetics, 76, 887–893. 10.1086/429864 15789306 PMC1199378

[ece35763-bib-0135] Zhuang, L. , Ye, Z. , & Zhang, C. (2015). Application of otolith shape analysis to species separation in *Sebastes* spp. From the Bohai Sea and the Yellow Sea, northwest Pacific. Environmental Biology of Fishes, 98, 547–558. 10.1007/s10641-014-0286-z

