# Supplementary material for: Sex matters: Otolith shape and genomic variation in deacon rockfish (Sebastes diaconus)
Source: Ecol Evol. 2019 Nov 7;9(23):13153–73. doi: 10.1002/ece3.5763 (PMC6912905; doi:10.1002/ece3.5763)

Appendix S1: Supplementary Tables and Figures

SUPPLEMENTARY TABLES

**Table S1**

The 96 rockfish individuals, 6 quality control repeats and 3 negative controls (no template DNA) processed for RAD sequencing. The sequencing plate, sample site and sex are provided for each individual. The table specifies which individuals were used for each dataset in the genetic analysis.

**Table S2**

Initial sequencing results for each of the three plates (library indices of 36 specimens) on the Illumina Hi-Seq 3000 platform, and reads removed during quality control using the *process_radtags* component of stacks 1.47.

**Table S3**

Results of varying certain parameters in *ustacks, cstacks* and *populations* components of stacks 1.47. Complete lists of parameters used for each component are provided in the methods section of the main text. Putative paralogous sequence variants (PSVs) and loci in linkage disequilibrium (LD) were not estimated and removed for these trial runs. Highlighted rows were settings used for final analyses presented in the main text.

**Table S4**

Mean Wang relatedness and variance within and among sample sites tested in the loci dataset comparing the three sample sites using 73 deacon rockfish (15,371 loci).

**Table S5**

Pairwise F_ST_ values (Weir & Cockerham, 1984) estimated using the stampp R package for the loci datasets comparing the three sample sites and the nearshore and offshore groups when the 92 putatively adaptive, sex-linked loci are removed. Estimated 95% confidence intervals (in parentheses) and p-values are listed below each F_ST_ estimate (using 5000 bootstraps). Statistically significant results are marked with an asterisk (*).

**Table S1**

| **Specimen ID** | **Plate** | **Sample**  **site** | **Nearshore**  **vs**  **offshore** | | **Three**  **sites** | **Sex** | **Female**  **three**  **sites** | **Male**  **three**  **sites** |
| --- | --- | --- | --- | --- | --- | --- | --- | --- |
| Sil17_001 | 1 | Siletz Reef |  | |  |  |  |  |
| Sil17_001_QC | 2 | Siletz Reef | Y | | Y | F | F |  |
| Sil17_002 | 1 | Siletz Reef | Y | | Y | F | F |  |
| Sil17_003 | 1 | Siletz Reef | Y | | Y | F | F |  |
| Sil17_004 | 2 | Siletz Reef | Y | | Y | F | F |  |
| Sil17_005 | 2 | Siletz Reef | Y | | Y | F | F |  |
| Sil17_006 | 2 | Siletz Reef | Y | | Y | F | F |  |
| Sil17_007 | 3 | Siletz Reef | Y | | Y | F | F |  |
| Sil17_008 | 3 | Siletz Reef | Y | | Y | F | F |  |
| Sil17_009 | 1 | Siletz Reef | Y | | Y | F | F |  |
| Sil17_010 | 1 | Siletz Reef | Y | | Y |  |  |  |
| Sil17_011 | 1 | Siletz Reef | Y | | Y | F | F |  |
| Sil17_012 | 2 | Siletz Reef | Y | | Y | M |  | M |
| Sil17_013 | 2 | Siletz Reef | Y | | Y | F | F |  |
| Sil17_014 | 2 | Siletz Reef | Y | | Y | M |  | M |
| Sil17_015 | 3 | Siletz Reef | Y | | Y | F | F |  |
| Sil17_016 | 3 | Siletz Reef | Y | | Y |  |  |  |
| Sil17_017 | 1 | Siletz Reef | Y | | Y | M |  | M |
| Sil17_018 | 1 | Siletz Reef | Y | | Y | M |  | M |
| Sil17_019 | 1 | Siletz Reef |  | |  |  |  |  |
| Sil17_019_QC | 3 | Siletz Reef | Y | | Y | M |  | M |
| Sil17_020 | 2 | Siletz Reef | Y | | Y | M |  | M |
| Sil17_021 | 2 | Siletz Reef | Y | | Y | F | F |  |
| Sil17_022 | 2 | Siletz Reef | Y | | Y | M |  | M |
| Sil17_024 | 3 | Siletz Reef | Y | | Y | F | F |  |
| Sil17_025 | 1 | Siletz Reef | Y | | Y | F | F |  |
| Sil17_026 | 1 | Siletz Reef | Y | | Y | F | F |  |
| Sea17_076 | 2 | Seal Rock | Y | | Y | F | F |  |
| Sea17_077 | 2 | Seal Rock | Y | | Y | F | F |  |
| Sea17_078 | 2 | Seal Rock | Y | | Y | F | F |  |
| Sea17_079 | 3 | Seal Rock | Y | | Y | M |  | M |
| Sea17_080 | 3 | Seal Rock | Y | | Y | F | F |  |
| Sea17_081 | 1 | Seal Rock | Y | | Y | M |  | M |
| Sea17_082 | 1 | Seal Rock | Y | | Y | M |  | M |
| Sea17_083 | 1 | Seal Rock | Y | | Y | M |  | M |
| Sea17_084 | 2 | Seal Rock | Y | | Y | F | F |  |
| Sea17_085 | 2 | Seal Rock | Y | | Y | F | F |  |
| Sea17_086 | 2 | Seal Rock | Y | | Y | F | F |  |
| Sea17_087 | 3 | Seal Rock | Y | | Y | F | F |  |
| Sea17_088 | 3 | Seal Rock | Y | | Y | M |  | M |
| Sea17_089 | 3 | Seal Rock | Y | | Y | F | F |  |
| Sea17_091 | 3 | Seal Rock | Y | | Y | F | F |  |
| Sea17_091_QC | 1 | Seal Rock |  | |  |  |  |  |
| Sea17_092 | 3 | Seal Rock | Y | | Y | F | F |  |
| Sea17_093 | 3 | Seal Rock | Y | | Y | M |  | M |
| Sea17_095 | 3 | Seal Rock | Y | | Y | F | F |  |
| Sea17_096 | 3 | Seal Rock | Y | | Y | F | F |  |
| Sea17_097 | 3 | Seal Rock | Y | | Y | F | F |  |
| Sea17_098 | 3 | Seal Rock | Y | | Y | F | F |  |
| Sea17_099 | 3 | Seal Rock | Y | | Y | F | F |  |
| Sea17_100 | 3 | Seal Rock | Y | | Y | F | F |  |
| Sto17_027 | 1 | Stonewall Bank | Y | |  | F |  |  |
| Sto17_028 | 2 | Stonewall Bank | Y | |  | F |  |  |
| Sto17_029 | 2 | Stonewall Bank | Y | |  | F | F |  |
| Sto17_030 | 2 | Stonewall Bank | Y | |  | F |  |  |
| Sto17_031 | 3 | Stonewall Bank | Y | |  | F | F |  |
| Sto17_032 | 3 | Stonewall Bank | Y | |  | F | F |  |
| Sto17_033 | 1 | Stonewall Bank | Y | |  | F | F |  |
| Sto17_034 | 1 | Stonewall Bank | Y | |  | F | F |  |
| Sto17_035 | 1 | Stonewall Bank | Y | | Y | F |  |  |
| Sto17_036 | 2 | Stonewall Bank | Y | | Y | F |  |  |
| Sto17_037 | 2 | Stonewall Bank | Y | |  | F | F |  |
| Sto17_037_QC | 1 | Stonewall Bank |  | |  |  |  |  |
| Sto17_038 | 2 | Stonewall Bank | Y | |  | M |  | M |
| Sto17_039 | 3 | Stonewall Bank | Y | |  | F | F |  |
| Sto17_040 | 3 | Stonewall Bank | Y | | Y | M |  | M |
| Sto17_041 | 1 | Stonewall Bank | Y | | Y | F |  |  |
| Sto17_042 | 1 | Stonewall Bank | Y | |  | F | F |  |
| Sto17_043 | 1 | Stonewall Bank | Y | | Y | F |  |  |
| Sto17_044 | 2 | Stonewall Bank | Y | | Y | F | F |  |
| Sto17_045 | 2 | Stonewall Bank | Y | |  | M |  |  |
| Sto17_046 | 2 | Stonewall Bank | Y | | Y | F |  |  |
| Sto17_047 | 3 | Stonewall Bank | Y | | Y | M |  |  |
| Sto17_048 | 3 | Stonewall Bank | Y | | Y | M |  | M |
| Sto17_049 | 1 | Stonewall Bank | Y | |  | F | F |  |
| Sto17_050 | 1 | Stonewall Bank | Y | | Y | F |  |  |
| Sto17_051 | 1 | Stonewall Bank | Y | |  | F |  |  |
| Sto17_052 | 2 | Stonewall Bank | Y | | Y | M |  | M |
| Sto17_053 | 2 | Stonewall Bank | Y | |  | F | F |  |
| Sto17_054 | 2 | Stonewall Bank | Y | | Y | F |  |  |
| Sto17_055_QC | 2 | Stonewall Bank |  | |  |  |  |  |
| Sto17_056 | 3 | Stonewall Bank | Y | |  | M |  |  |
| Sto17_057 | 1 | Stonewall Bank | Y | | Y | M |  |  |
| Sto17_058 | 1 | Stonewall Bank | Y | | Y | M |  |  |
| Sto17_059 | 1 | Stonewall Bank | Y | |  | F |  |  |
| Sto17_060 | 2 | Stonewall Bank | Y | |  | F |  |  |
| Sto17_061 | 2 | Stonewall Bank | Y | | Y | F |  |  |
| Sto17_062 | 2 | Stonewall Bank | Y | | Y | F | F |  |
| Sto17_063 | 3 | Stonewall Bank | Y | | Y | F |  |  |
| Sto17_064 | 3 | Stonewall Bank | Y | | Y | F | F |  |
| Sto17_065 | 1 | Stonewall Bank | Y | |  | F |  |  |
| Sto17_066 | 1 | Stonewall Bank | Y | | Y | M |  | M |
| Sto17_067 | 1 | Stonewall Bank | Y | | Y | M |  | M |
| Sto17_068 | 2 | Stonewall Bank | Y | | Y | F |  |  |
| Sto17_069 | 2 | Stonewall Bank | Y | |  | F |  |  |
| Sto17_070 | 2 | Stonewall Bank | Y | | Y | F | F |  |
| Sto17_071 | 3 | Stonewall Bank | Y | | Y | F | F |  |
| Sto17_072 | 3 | Stonewall Bank | Y | |  | F | F |  |
| Sto17_073 | 1 | Stonewall Bank | Y | |  | M |  |  |
| Sto17_073_QC | 3 | Stonewall Bank |  | |  |  |  |  |
| Sto17_074 | 1 | Stonewall Bank | Y | |  | M |  | M |
| Sto17_075 | 1 | Stonewall Bank | Y | |  | F |  |  |
| NC1 | 1 |  |  | |  |  |  |  |
| NC2 | 2 |  |  | |  |  |  |  |
| NC3 | 3 |  |  | |  |  |  |  |
| **Dataset sample totals** | | | | **96** | **73** | **94** | **50** | **20** |

**Table S2**

| **Counts** | **Plate 1** | **Plate 2** | **Plate 3** | *Totals* |
| --- | --- | --- | --- | --- |
| **Total pairs of reads** | 106,605,555 | 126,092,588 | 110,003,961 | *342,702,104* |
| **Total single reads**  **(R1 and R2)** | 213,211,110 | 252,185,176 | 220,007,922 | *685,404,208* |
| **Ambiguous barcode drops** | 120,689,032 | 140,416,681 | 120,468,281 | *381,573,994* |
| **Low quality read drops** | 28,483 | 34,561 | 31,221 | *94,265* |
| **Ambiguous RAD-tag drops** | 4,531,364 | 5,183,844 | 2,954,376 | *12,669,584* |
| **Retained pairs of reads** | 87,962,231 | 106,550,090 | 96,554,044 | *291,066,365* |
| **Percentage pairs retained** | 82.5% | 84.5% | 87.8% | *84.9%* |

**Table S3**

| **# indiv.** | **# pop.** | **ustacks** | **cstacks** | **populations** | **# variant and invariant loci** | **# variant loci** |
| --- | --- | --- | --- | --- | --- | --- |
| 73 | 3 | -m 2 -M 2 -N 4 | -n 1 | -m 5 -p 3 -r 0.6 | 18,277 | 15,691 |
| 73 | 3 | -m 2 -M 2 -N 4 | -n 1 | -m 10 -p 3 -r 0.6 | 14,960 | 12,489 |
| 73 | 3 | -m 2 -M 2 -N 4 | -n 2 | -m 5 -p 3 -r 0.6 | 18,532 | 15,975 |
| 73 | 3 | -m 2 -M 3 -N 5 | -n 1 | -m 5 -p 3 -r 0.6 | 18,541 | 16,027 |
| 96 | 2 | -m 2 -M 2 -N 4 | -n 1 | -m 5 -p 2 -r 0.6 | 17,194 | 16,266 |
| 96 | 2 | -m 2 -M 2 -N 4 | -n 1 | -m 10 -p 2 -r 0.6 | 15,301 | 14,115 |
| 96 | 2 | -m 2 -M 2 -N 4 | -n 2 | -m 5 -p 2 -r 0.6 | 17,495 | 16,310 |
| 96 | 2 | -m 2 -M 3 -N 5 | -n 1 | -m 5 -p 2 -r 0.6 | 17,480 | 16,321 |

**Table S4**

|  |  |  |  |
| --- | --- | --- | --- |
| **Sample**  **sites** | **Siletz Reef** | **Seal Rock** | **Stonewall Bank** |
| **Siletz Reef** | -0.0213  0.0014 |  |  |
| **Seal Rock** | -0.0365  0.0009 | -0.0454  0.0004 |  |
| **Stonewall Bank** | -0.0264  0.0010 | -0.3783  0.0006 | -0.0263  0.0007 |

**Table S5**

|  |  |  |  |  |  |  |  |
| --- | --- | --- | --- | --- | --- | --- | --- |
| **#**  **indiv.** | **#**  **pop.** | **Loci**  **dataset** | **# loci** | **Siletz Reef vs**  **Seal Rock** | **Siletz Reef vs**  **Stonewall Bank** | **Seal Rock vs**  **Stonewall Bank** | **Nearshore vs**  **Offshore** |
| Three populations (Siletz Reef, Seal Rock, Stonewall Bank) | | | | | | | |
| 73 | 3 | Neutral with sex-linked loci removed | 15,281 | 0.0014  (0.0006 – 0.0021)  p = <0.0001  * | 0.0010  (0.0003 – 0.0016)  p = 0.0016  * | 0.0011  (0.0004 – 0.0017)  p = 0.0004  * |  |
| Two populations (Nearshore vs Offshore) | | | | | | | |
| 96 | 2 | Neutral with sex-linked loci removed | 15,845 |  |  |  | 0.0005  (0.0001 – 0.0008)  p = 0.0042  * |

SUPPLEMENTARY FIGURES

**Fig. S1**

Histograms for the age of sex identified otolith samples for each sample site (N = 668).

**Fig. S2**

A bar graph showing coverage depth per individual for the nearshore vs offshore dataset (N = 96), for the catalog produced by the *denovo* pipeline in stacks 1.47. Mean coverage depth per individual is shown in blue (with standard deviation shown by error bars in black), and maximum coverage per individual is shown in yellow. See the main text Methods for the parameters applied.

**Fig. S3**

Probability density functions for each of the 1000 iterations for the nearshore and offshore collections with different sample sizes (gray boxes on Y axis). Values in the gray boxes on Y axes denote the number of otoliths (equal numbers between nearshore and offshore) sampled to generate the distribution.

**Fig. S4**

Average probability density functions for the combined 1000 iterations for the nearshore and offshore collections with different sample sizes (gray boxes on Y axis). Values in the gray boxes on Y axes denote the number of otoliths (equal numbers between nearshore and offshore) sampled to generate the distribution.

**Fig. S5**

F-statistic values from the PERMANOVA test for the different sample sizes used to test for evidence of differences between different otolith sample sizes. Each sample size was calculated using 1000 iterations of sampling with replacement.

**Fig. S6**

Plots produced using the whoa R package, used to examine the distribution of genotypes in the loci datasets comparing the three samples sites and the nearshore and offshore groups. The top row of figures (A, C) are scatterplots showing the frequency of genotypes (0 = homozygote allele A, 1 = heterozygote, 2 = homozygote allele B) in loci datasets. Lines drawn show the expected trend and bounds for genotypic frequencies based on Hardy-Weinberg equilibrium. The bottom row of figures (B, D) shows the relationship between estimated heterozygote miscall rate versus mean coverage depth, with loci in each dataset organized into bins based on mean coverage depth.

1. Genotype frequencies in the loci dataset comparing the three sample sites (n = 73, 15,371 loci).
2. Genotype frequencies in the loci dataset comparing the nearshore and offshore groups (N = 96, 15,937 loci).
3. Estimated heterozygote miscall rate versus mean coverage depth for the loci dataset comparing the three sample sites (n = 73, 15,371 loci).
4. Estimated heterozygote miscall rate versus mean coverage depth for the loci dataset comparing the nearshore and offshore groups (N = 96, 15,937 loci).

**Fig. S7**

Plots produced using the whoa R package, used to examine the distribution of genotypes in the loci dataset comparing males and females.

1. A scatterplot showing the frequency of genotypes (0 = homozygote allele A, 1 = heterozygote, 2 = homozygote allele B) in the loci dataset comparing males and females (n = 94, 15,657 loci). Lines drawn show the expected trend and bounds for genotypic frequencies based on Hardy-Weinberg equilibrium.
2. A plot showing the relationship between estimated heterozygote miscall rate versus mean coverage depth in the loci dataset comparing males and females (n = 94, 15,657 loci). Loci are organized into bins based on mean coverage depth.

**Fig. S8**

Plots produced using the whoa R package, used to examine the distribution of genotypes in the male and female-only loci datasets. The top row of figures (A, C) are scatterplots showing the frequency of genotypes (0 = homozygote allele A, 1 = heterozygote, 2 = homozygote allele B) in loci datasets. Lines drawn show the expected trend and bounds for genotypic frequencies based on Hardy-Weinberg equilibrium. The bottom row of figures (B, D) show the relationship between estimated heterozygote miscall rate versus mean coverage depth, with loci in each dataset organized into bins based on mean coverage depth.

1. Genotype frequencies in the loci dataset comparing only females from the three sample sites (n = 50, 14,678 loci).
2. Genotype frequencies in the loci dataset comparing only males from the three sample sites (n = 20, 14,564 loci).
3. Estimated heterozygote miscall rate versus mean coverage depth for the loci dataset comparing only females from the three sample sites (n = 50, 14,678 loci).
4. Estimated heterozygote miscall rate versus mean coverage depth for the loci dataset comparing only males from the three sample sites (n = 20, 14,564 loci).

**Fig. S9**

Scatterplots presenting genetic variation among deacon rockfish individuals as estimated by principal components analysis (PCA), based on the RAD sequenced SNP genotype data. Sample sizes and the coloration of individuals by potential population is explained in the key, although it should be remembered that PCA does not test for differences among groups - it only visualizes variance among samples.

1. SNP variation among males in a dataset comparing sampling from the three sample sites only (n = 20). The first two PCs presented represent 5.9% and 5.8% of variation among individuals respectively.
2. SNP variation among females in a dataset comparing sampling from the three sample sites only (n = 50). The first two PCs presented represent 2.5% and 2.4% of variation among individuals respectively.

**Fig. S10**

Two scatterplots comparing F_ST_ and observed heterozygosity (H_O_) for loci in the sex comparison dataset (n = 94), produced using the fsthet R package. The top and bottom red lines show the smoothed quantiles of the dataset. Loci identified as outliers by fsthet occur in the plotted areas outside of the quantiles.

**Fig. S11**

Mean K probability (LK) plots for each genetic dataset: A) three sites, B) two groups, and C) sex. For sex, putatively adaptive and presumed neutral loci are separated. Values of K (the true number of clusters) with the highest mean K probability for each dataset were chosen as the optimal number of clusters for each dataset.

**Fig. S12**

DeltaK plots for each genetic dataset: A) three sites, B) two groups, and C) sex. For sex, putatively adaptive and presumed neutral loci are separated. Values of K (the true number of clusters) with the highest DeltaK value for each dataset were chosen as the optimal number of clusters for each dataset.

**Fig. S13**

Mean K probability (LK) plots for each genetic dataset: A) female-only three site and B) male-only three sites. Values of K with the highest mean K probability for each dataset were chosen as the optimal number of clusters for each dataset.

**Fig. S14**

DeltaK plots for each genetic dataset: A) female-only three site and B) male-only three sites. Values of K with the highest DeltaK value for each dataset were chosen as the optimal number of clusters for each dataset.

**Fig. S1**


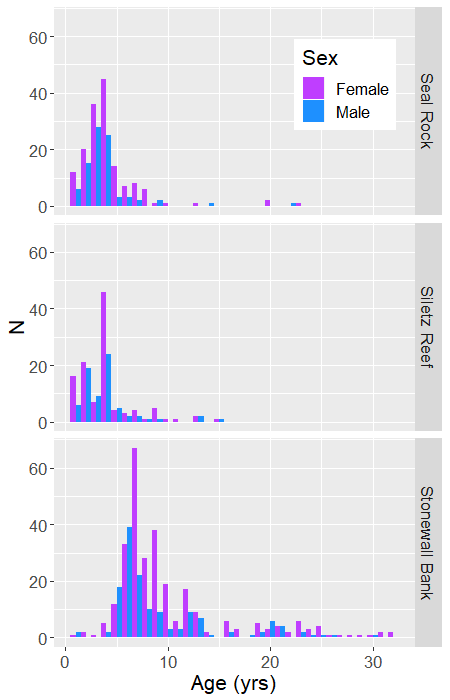


**Fig. S2**


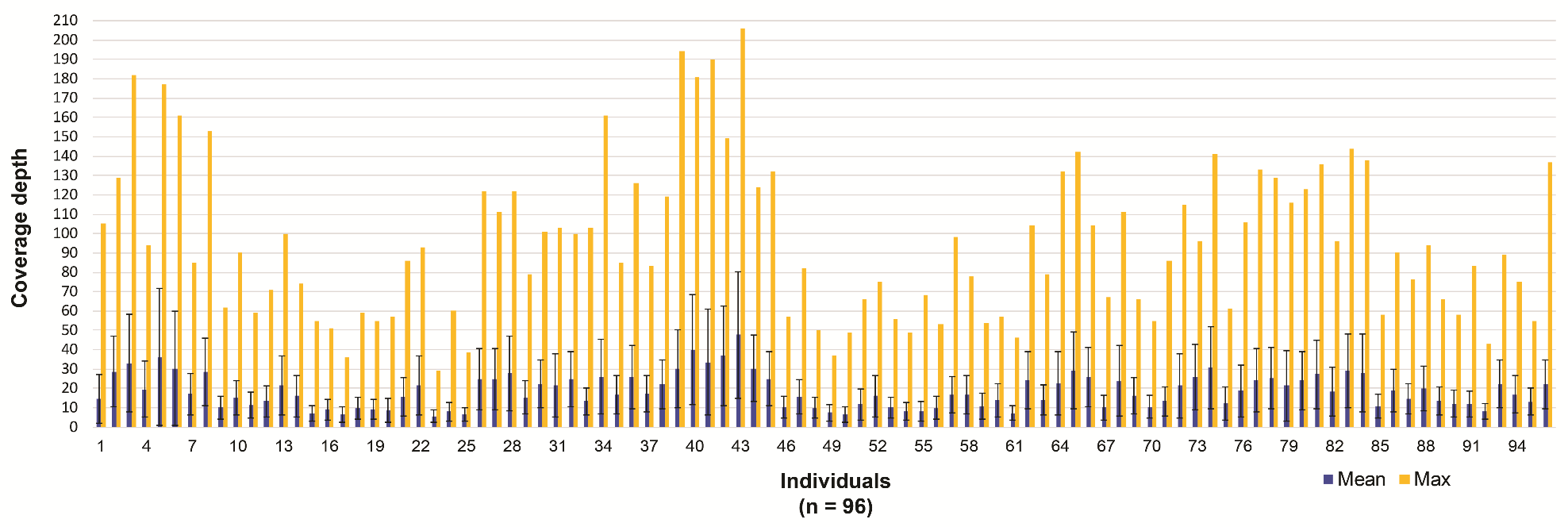


**Fig. S3**


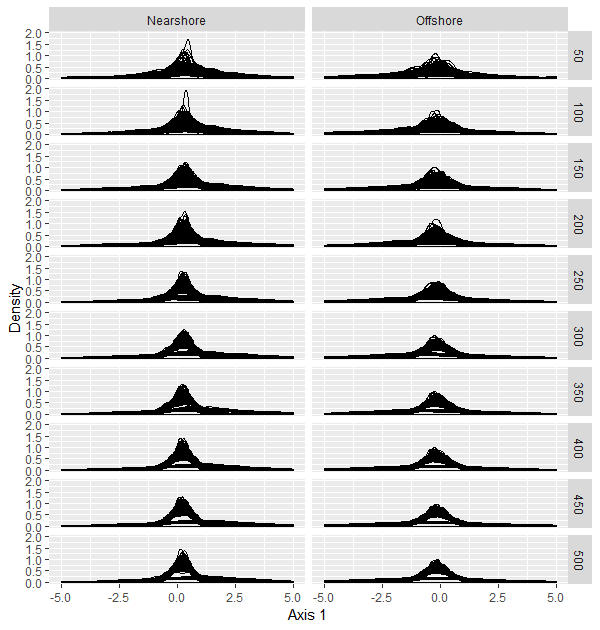


**Fig. S4**


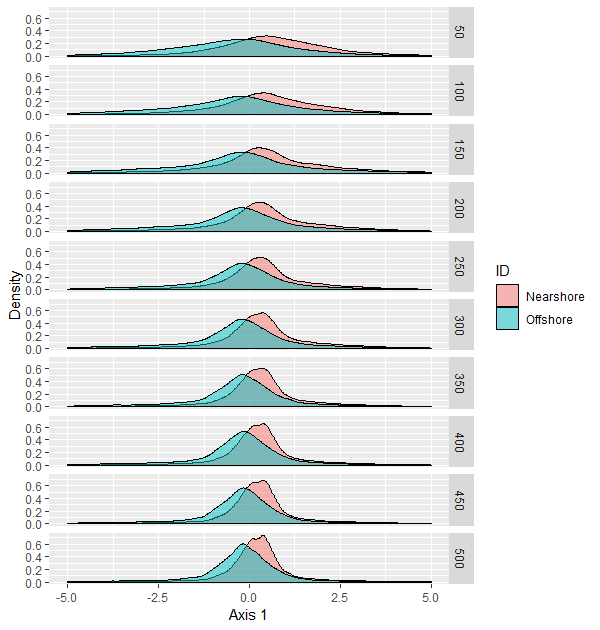


Fig. A5


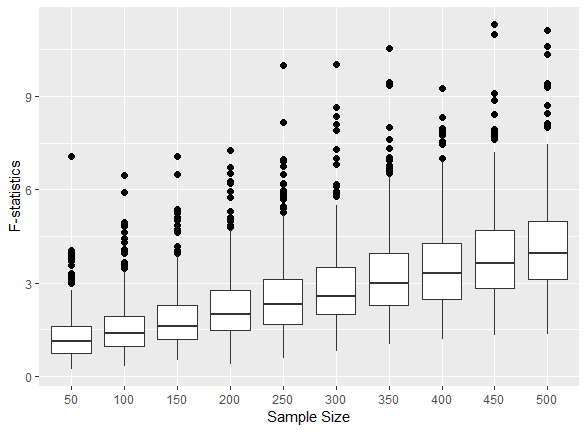


**Fig. S6**


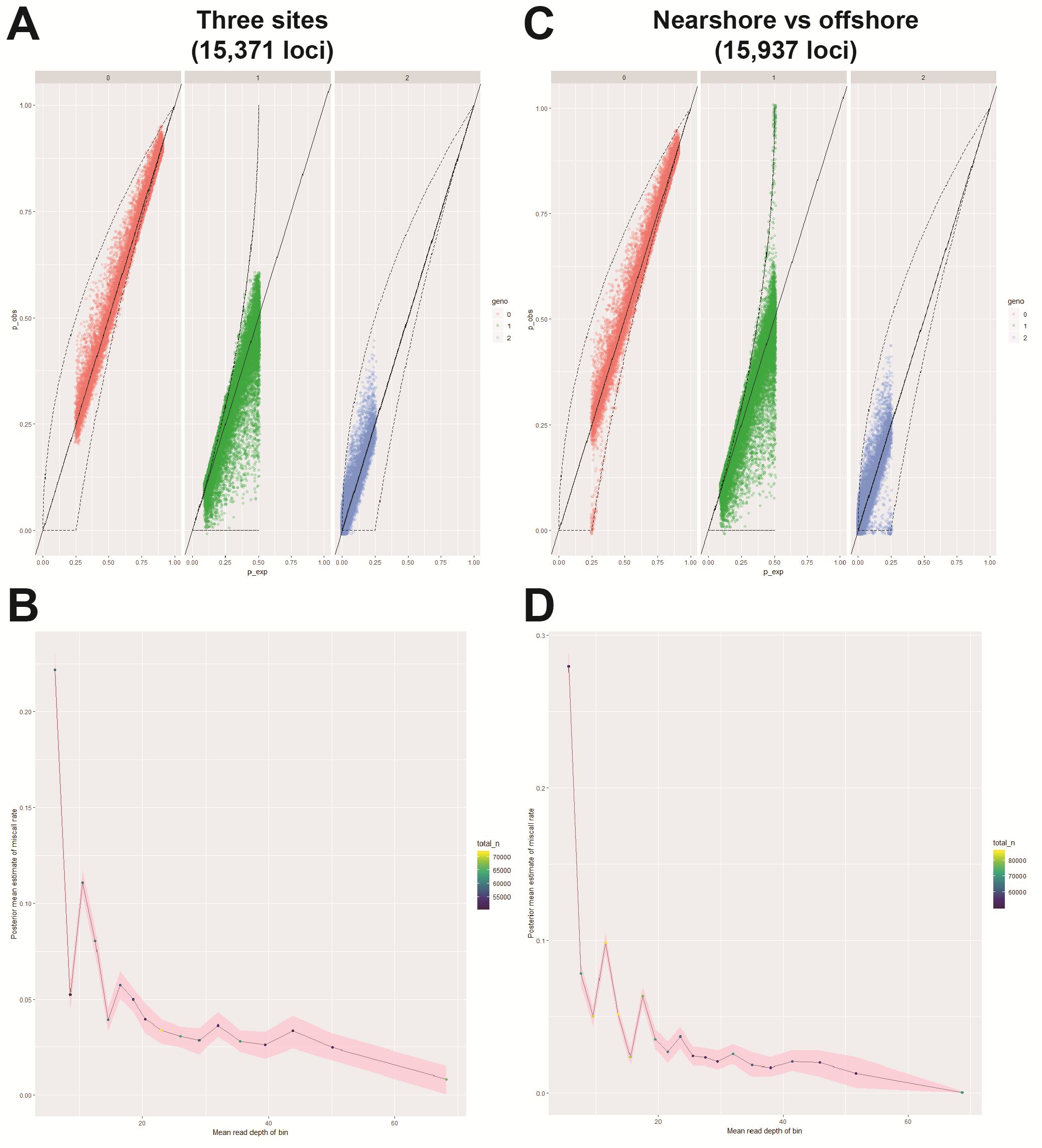


**Fig. S7**


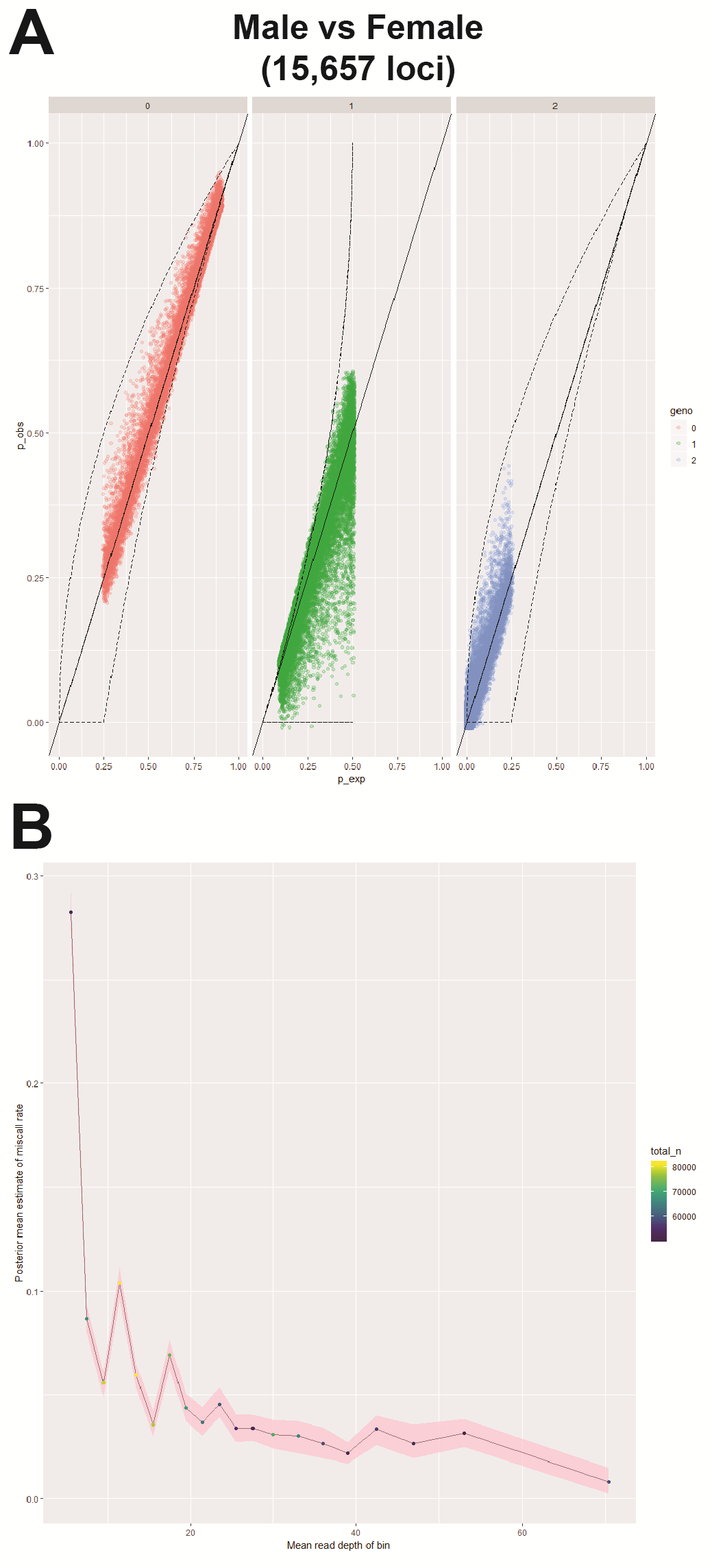


**Fig. S8**


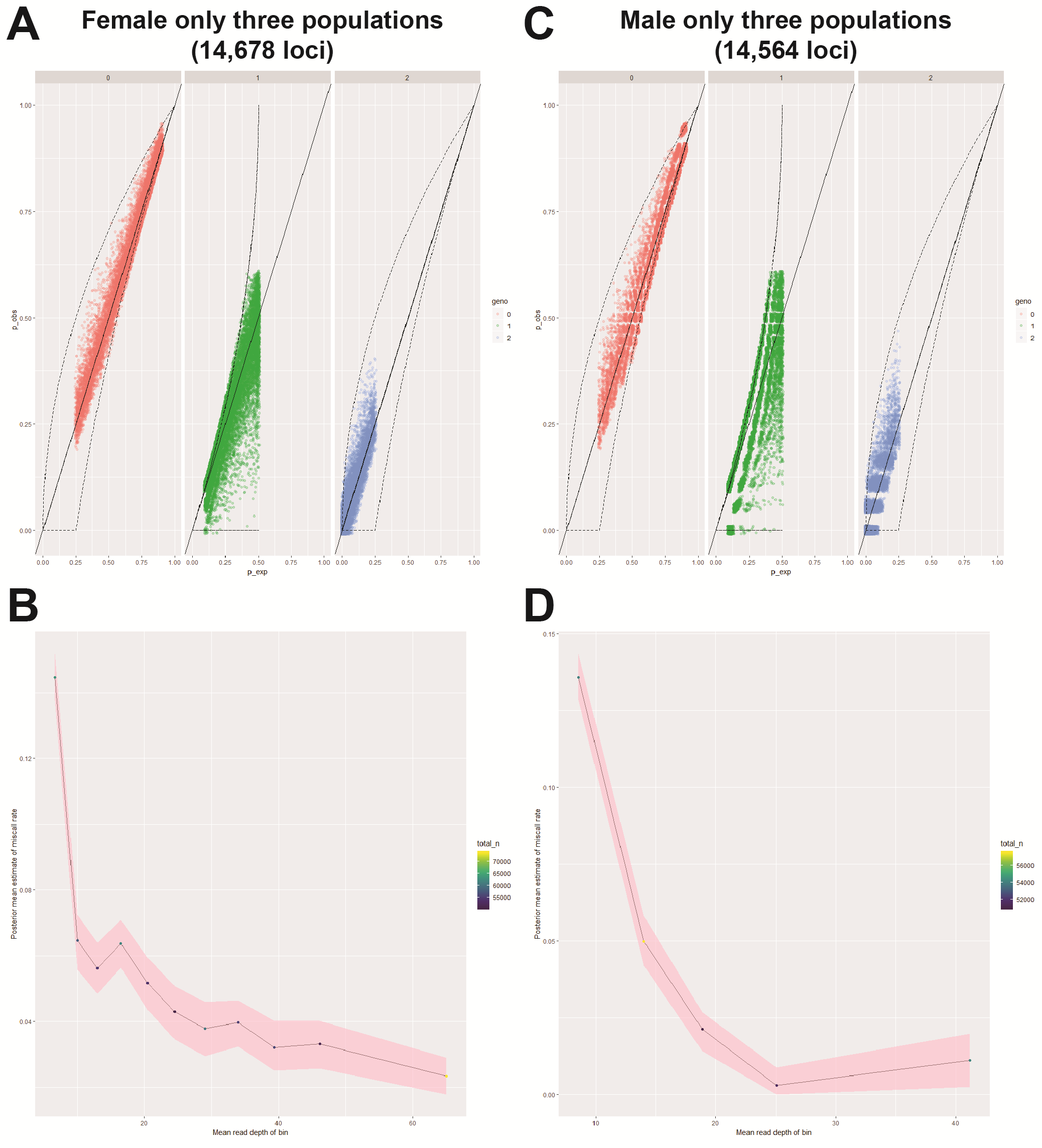


**Fig. S9**


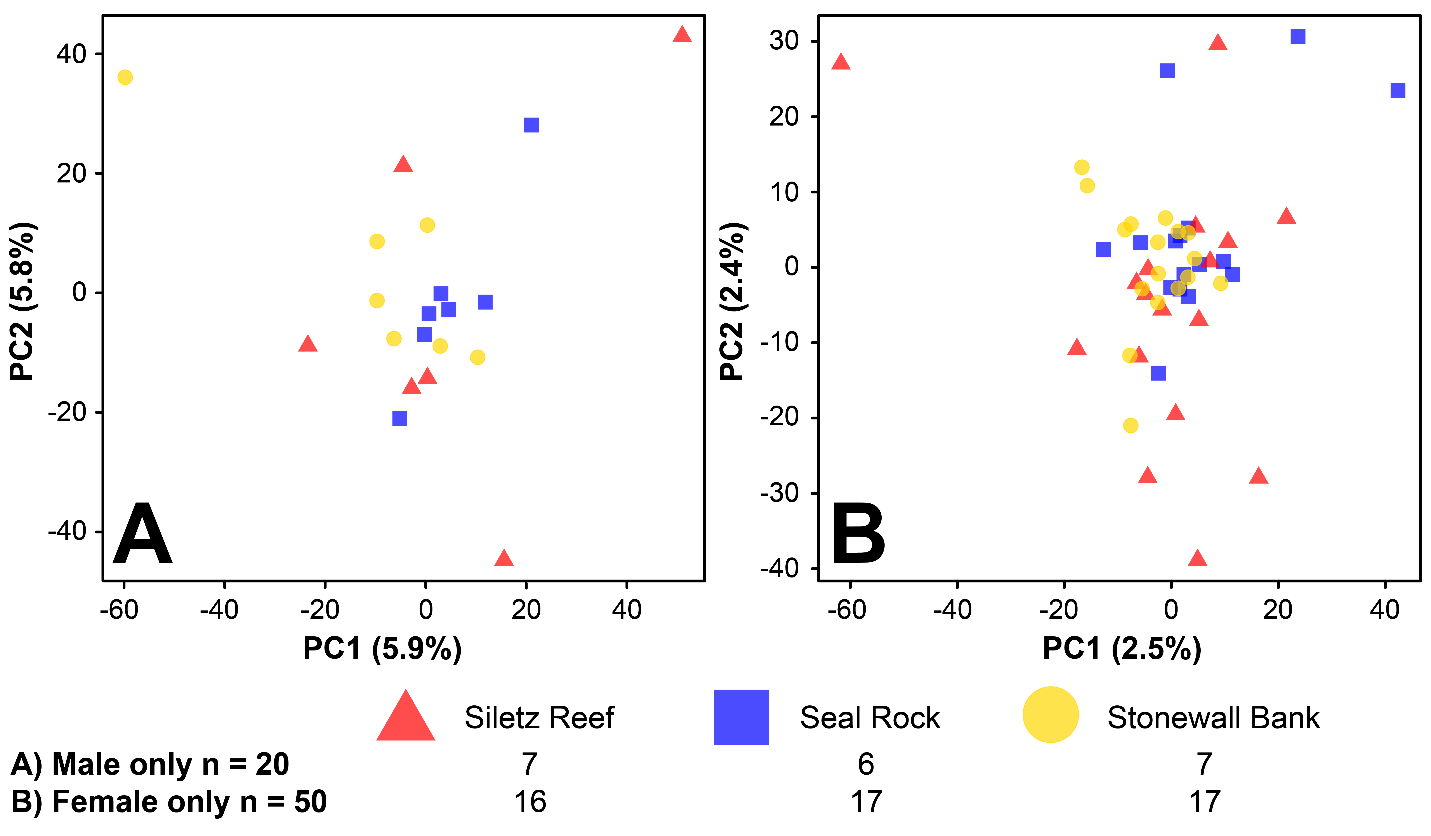


**Fig. S10**


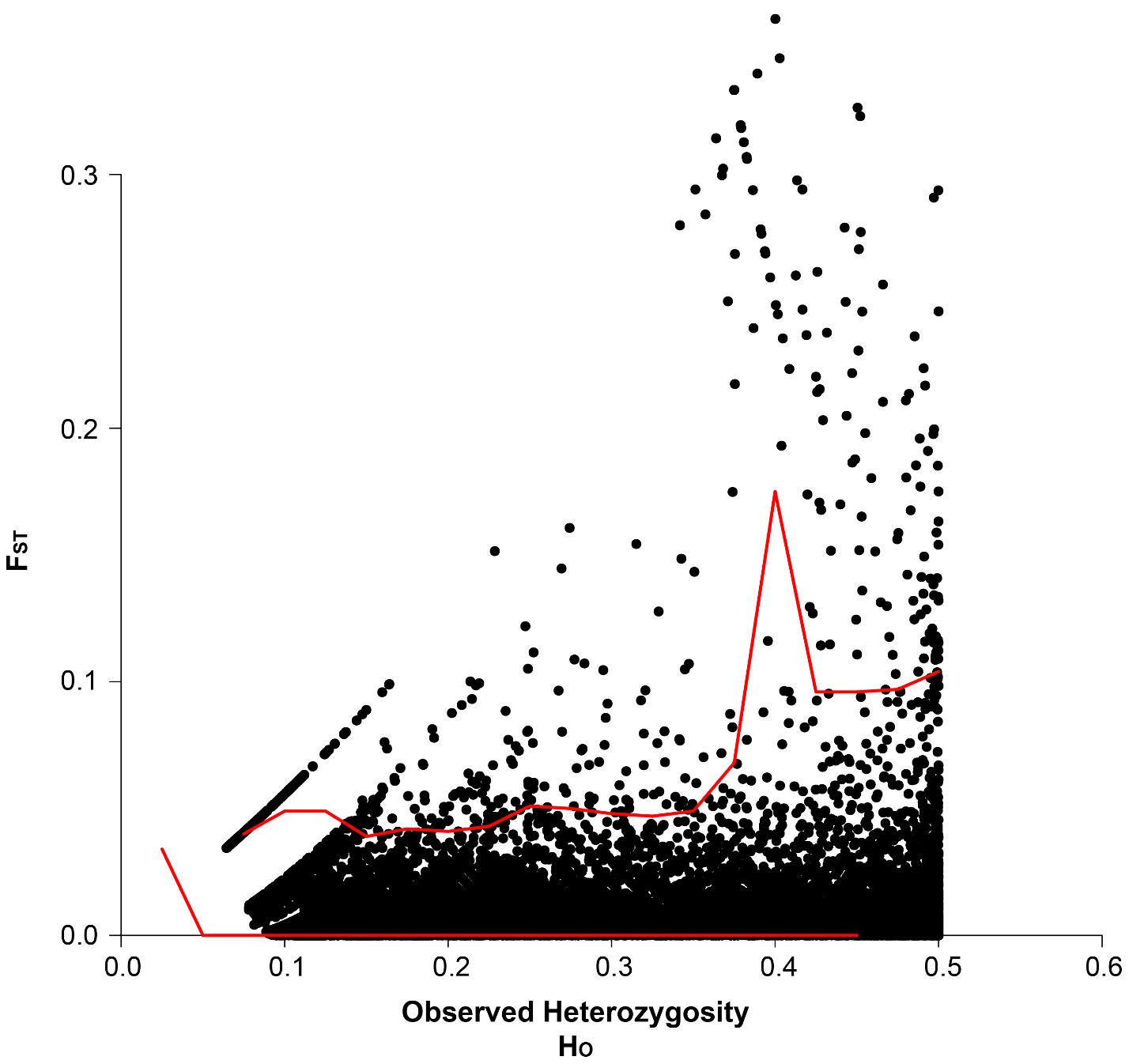


**Fig. S11**

**
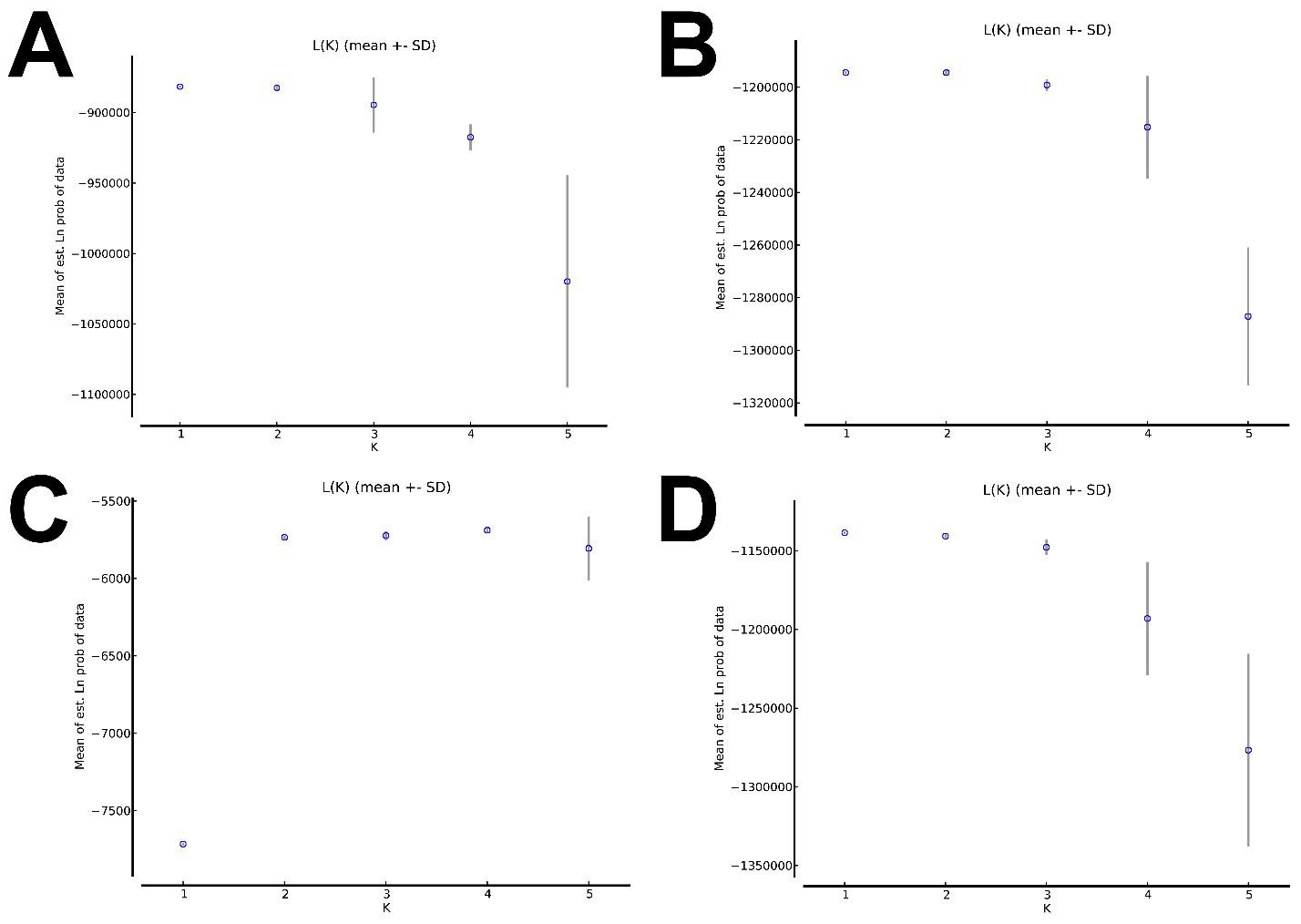
**

**Fig. S12**

**
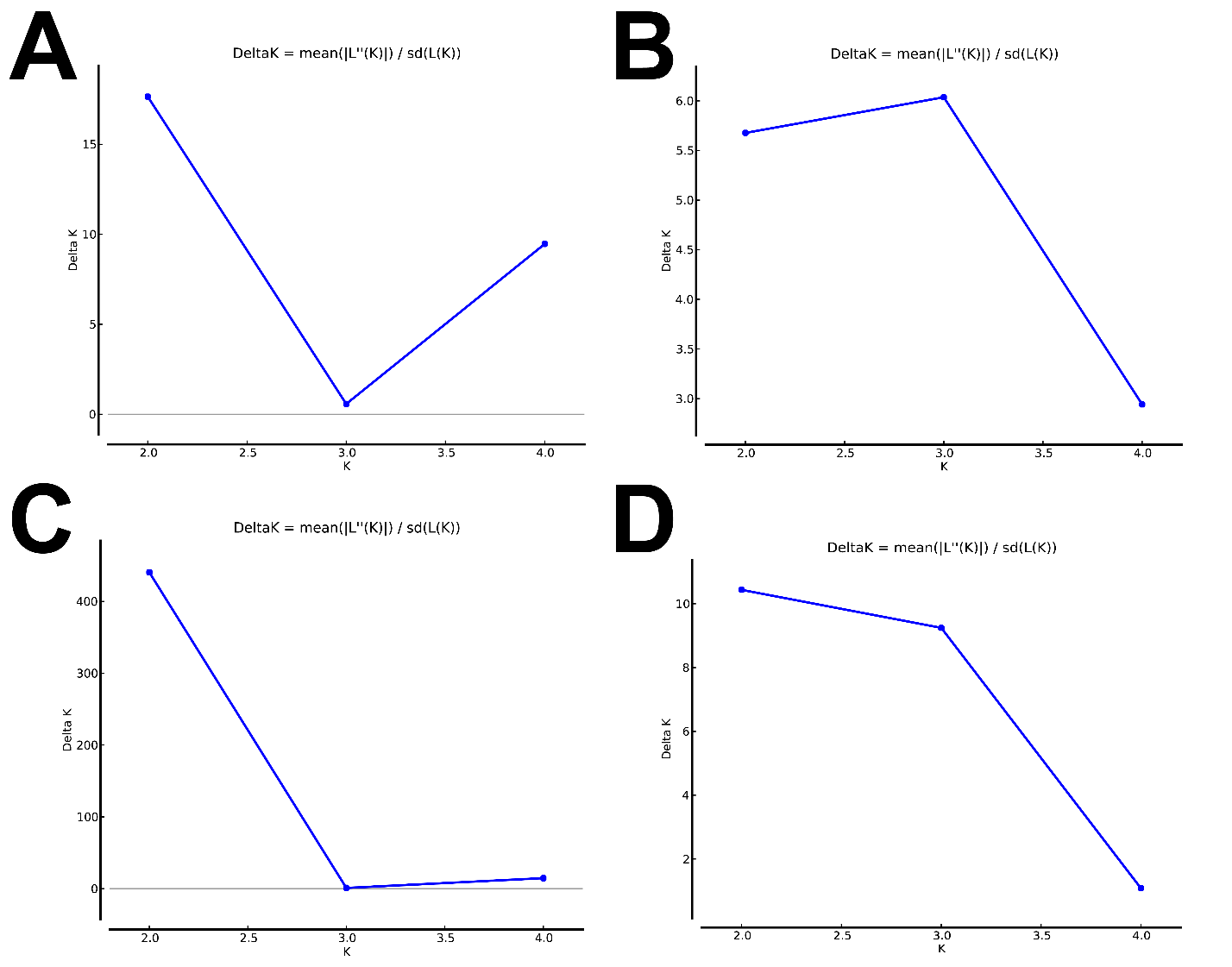
**

**Fig. S13**

**
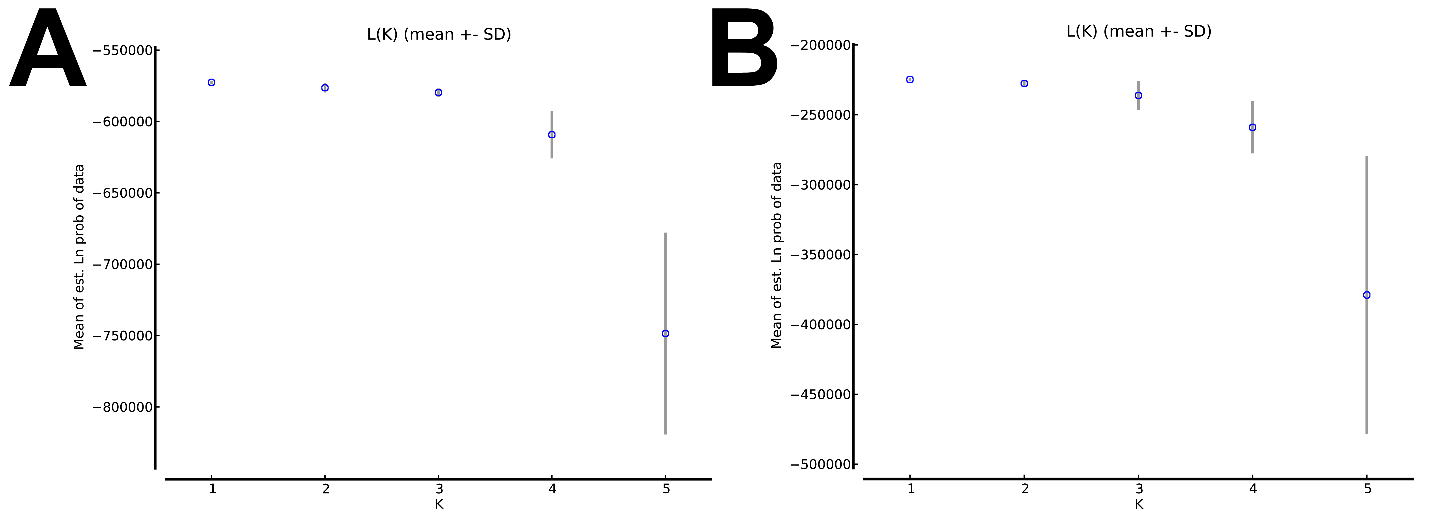
**

**Fig. S14**


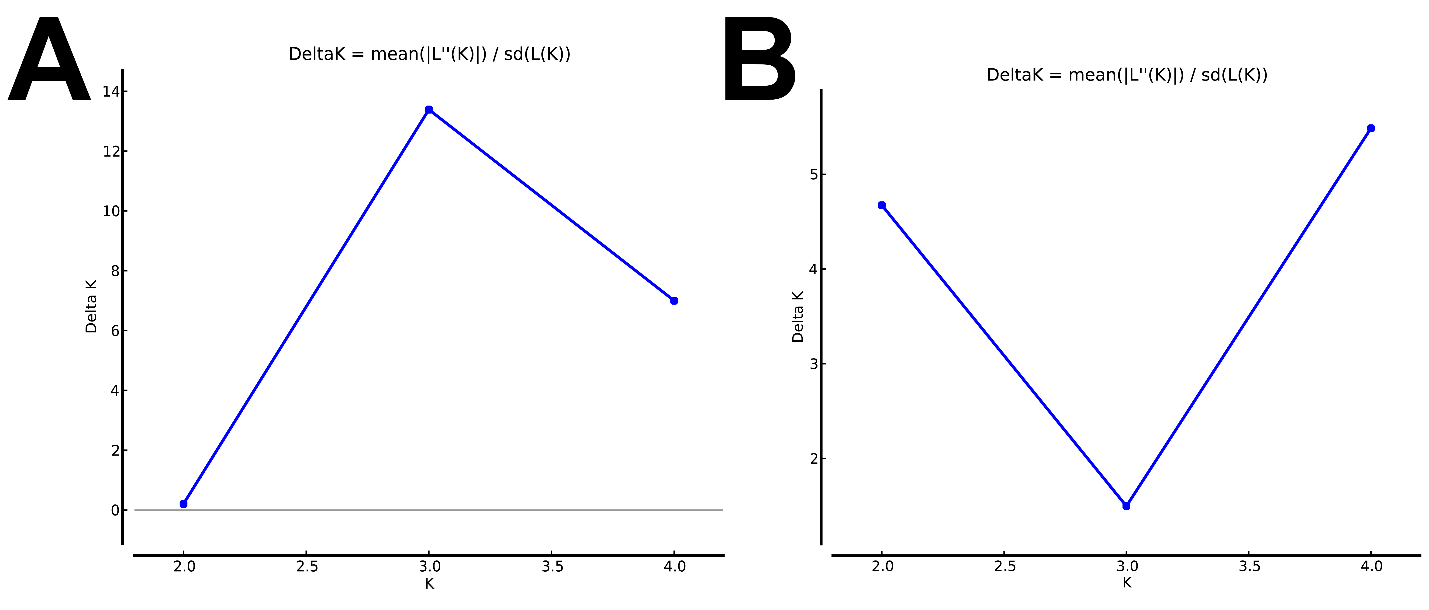

Supplement: Supplementary file 1 [file ECE3-9-13153-s001.docx]
